# Criteria, Greenhouse Gas, and Hazardous Air Pollutant Emissions Factors from Residential Cordwood and Pellet Stoves Using an Integrated Duty Cycle Test Protocol

**DOI:** 10.1021/acsestair.4c00135

**Published:** 2024-08-12

**Authors:** Nora Traviss, George Allen, Mahdi Ahmadi

**Affiliations:** † Northeast States Coordinated Air Use Management (NESCAUM), Boston, Massachusetts 02111, United States; ‡ Keene State College, Keene, New Hampshire 03435, United States; § University of North Texas, Denton, Texas 76203, United States

**Keywords:** residential wood heating, wood stoves, emissions, particulate matter, criteria air pollutants, hazardous air pollutants, greenhouse gases, metals

## Abstract

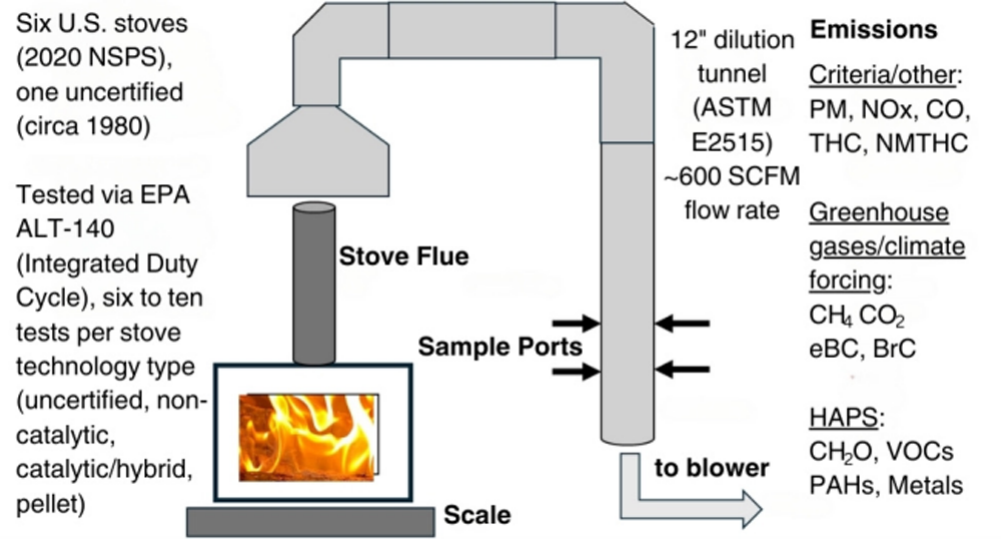

Air pollution from residential wood heating (RWH) presents challenges at the intersection of climate and public health. With a revised National Ambient Air Quality Standard (NAAQS, at 9 μg/m^3^) for particulate matter (PM) in the United States (U.S.), the Environmental Protection Agency (EPA) will likely classify new non-attainment areas due primarily to emissions from RWH. Agencies will use emissions factors (EFs) to develop attainment strategies. Many will rely on EPA modeling platforms based on data from the National Emissions Inventory (NEI). The NEI uses RWH EFs based on data from mid-1990’s in-situ studies and a speciation profile from a 2001 study of fireplace emissions. The NEI does not include greenhouse gas (GHG) emissions for this sector, which plays a key role when assessing climate reduction strategies for the buildings sector. Here, we tested seven wood stoves to determine EFs, representing various vintages and control technologies, using a novel test method that reflects in-use operational settings called the Integrated Duty Cycle. The study measured multiple pollutants concurrently: criteria pollutants (particulate matter [PM], CO, and NOx), nonmethane total hydrocarbons (NMTHCs), GHGs, black carbon (eBC), brown carbon (BrC), and multiple hazardous air pollutants (HAPs). We found no significant difference in PM EFs between uncertified and non-catalytic stove technologies. RWH EF results from this study exceeded 2020 NEI RWH EFs for NMTHC and multiple HAPs. Applying our study’s EFs to the 2020 NEI suggests that RWH, compared to all other sources, ranks as the 2nd largest source category of formaldehyde; the 3rd largest of benzene, 1,3-butadiene, and acrolein; and the 4th largest of Pb emissions. RWH also emits more methane compared to natural gas or oil residential heating, raising questions about substitution of wood as a climate neutral heating fuel. However, compared to uncertified stoves, pellet stove EFs (except toxic metals) were significantly lower (*p* < 0.01). In summary, RWH appears to be an underestimated source of PM (non-catalytic technology), methane, NMTHC, toxic metals, and other HAPs, which has important implications for climate and public health policy in the U.S. and globally.

## Abstract 2

This research utilized a novel stove operation and fueling test method to update decades-old pollutant emissions factors with new data from U.S. EPA-certified woodstoves. Compared to other categories of home heating, RWH is a substantial source of particulate matter, methane, lead, and multiple hazardous air pollutants, with implications for public health and climate in the U.S. and globally.

## 1 Introduction

Although air pollution from residential wood heating (RWH) presents global climate and public health challenges, many in the United States (U.S.) consider RWH to be a dependable, renewable, and lower cost approach to meet home heating needs. Approximately 11 million U.S. homes used wood or pellets for space heating in 2020.^1^ However, this low percentage (8.9%) of all American homes that burn wood for residential energy contributes up to 98% of fine particulate matter (PM_2.5_) from residential heating from all fuel types.^2,3^ Recent analysis confirms an increasing trend of RWH use in the Northeast, and that RWH is one of the top sources of PM_2.5_ emissions in all but eight states in the U.S.^2^ Estimating the total number of individuals in the United States exposed to woodsmoke from RWH is difficult, though in 2015 the estimate was 30 million people.^4^

The U.S. Environmental Protection Agency (EPA) estimates RWH emits approximately 485,000 tons per year of PM_2.5_ in the U.S. (the 4th largest source), a quantity greater than all on-road and non-road mobile PM_2.5_ emissions, combined (Figure S2).^2,5^ Decades of epidemiological research have linked PM_2.5_ exposure to cardiovascular morbidity and mortality.^6,7^ Researchers have estimated 10,000 to 40,000 premature mortalities occur annually in the U.S. due to exposure to RWH PM emissions.^8,9^ Recent epidemiological research has linked wildfire-PM exposure with multiple and varied negative health outcomes including incident dementia^10^ and asthma-related hospitalizations in older adults.^11^ Mehta et al.^12^ have reported increased risk of lung cancer in a U.S. cohort of women exposed to woodsmoke from fireplaces or wood stoves, including women who never smoked. Numerous adverse health effects from the organic and inorganic components of woodsmoke have been noted: elevated levels of cellular oxidative stress, DNA damage, and cytotoxicity *in vitro* and increased asthma symptoms, systemic inflammation, and levels of neutrophils in humans.^13−20^

Beyond PM_2.5_, RWH also emits nitrogen oxides (NOx), carbon monoxide (CO), greenhouse gases (GHGs), and compounds such as volatile organic compounds (VOCs), polycyclic aromatic hydrocarbons (PAHs), metals, and others. Fresh and aged VOCs and PAHs emitted by RWH can contribute to formation of secondary organic aerosols leading to additional impact on public health and climate.^21−23^ Certain hazardous air pollutants (HAPs) emitted by RWH, such as benzene and formaldehyde, pose elevated cancer risk to the public in urban and rural areas, with notable impact on low-income populations.^24^ RWH is also linked with higher levels of indoor PM and PAHs, increasing health risks to residents.^25−27^ Since RWH simultaneously contributes to indoor and outdoor air pollution, interventions to reduce RWH emissions would decrease both individual and population exposures.^28^ However, the number of co-pollutants emitted from RWH, the variety of appliance technology types in current use, and individual user behaviors challenge the development of effective policy to reduce emissions. Appliance technology and age of woodstoves in the U.S. vary from vintage cordwood stoves manufactured prior to the 1988 U.S. EPA New Source Performance Standards (NSPS) to much lower PM-emitting Step 2 NSPS pellet stoves. However, we note all wood heating systems emit far greater PM emissions than oil or natural gas home heating systems.^29^ While several studies have measured emissions from RWH appliances,^21−23,30−34^ these studies, on European or older U.S. stoves, have used highly varied operating protocols, wood moisture content, wood fuel species, or stove models not available in the U.S. retail market. The variability in research protocols and stove models makes comparison of emissions results across studies difficult, hindering efforts to reduce RWH pollution at scale in the U.S. and elsewhere.

Updated RWH emissions factors (EFs) from various generations of U.S. stove technologies, determined using a replicable stove operational protocol that more closely approximates “real-world” stove use, would contribute new RWH EF profiles for air quality research, modeling, planning, and policymaking for climate and public health. Residential energy emissions represent approximately 20% of GHG emissions in the U.S., but a follow-up detailed analysis of the carbon footprint of U.S. households categorized wood as “carbon-neutral”, a marginal energy source,^35^ and used a predicted, decreasing RWH market share to less than 2% by 2050. However, in some areas of the country like the Northeast U.S., RWH use is increasing.^2^ Evidence that brown carbon from local RWH aerosols contributes to near-UV light absorption and climate forcing is growing.^36,37^ Improved RWH emissions measurements of methane, black carbon, and brown carbon will assist scientists in understanding connections to climate impacts.

The most recent 2020 EPA National Emissions Inventory used RWH EFs for PM, CO, VOCs, and other pollutants from the 1996 EPA AP-42 Compilation of Air Pollutant Emissions Factors for residential wood stoves.^38,39^ AP-42 since has not incorporated potential technology improvements, especially for catalytic stoves, nor accounted for long term degradation of stove performance. Current approaches assume all certified stoves emit at similar levels. However, certified stoves could be more than 30 years old, well past the EPA estimated useful life of 20 years. Additionally, the EPA Office of the Inspector General (IG) reported multiple “flaws” with the RWH NSPS testing program, concluding that PM emissions from “certified” RWH may exceed PM certification limits,^40^ raising questions regarding the emissions performance from post-2015 NSPS newer stoves. The IG report also highlighted the environmental justice impacts of excess PM and HAPs from woodsmoke on tribal, low income, and minority populations.^40^ Many speciated compound RWH EFs, including HAP EFs, are based on a 2001 study of fireplace emissions,^41^ and since then, EPA researchers have recommended new RWH source emissions testing for the residential wood combustion category to update VOC and PM profiles.^42^

With the recent adoption of the lower annual PM_2.5_ National Ambient Air Quality Standard of 9 μg/m^3^, many more areas are expected to go into “non-attainment” due to RWH, including rural areas with no other major sources of PM. Updated RWH EFs could assist in evaluation of important policy interventions such as federal and state incentivized woodstove changeout programs to determine whether concurrent reductions in PM, HAPs, and GHGs have occurred. Many believe RWH changeout programs provide emission benefits.^28^ While rural Libby, MT is often cited as a successful woodstove changeout program that resulted in reductions of ambient and indoor PM_2.5_, the large scale of the changeout (replacing approximately 1100 stoves out of 2300 in a small community) was unique in the U.S.^28,43,44^ Additionally, Montana Department of Environmental Quality’s post-changeout review of Libby reported that approximately 18% of the 1100 woodstoves were replaced with alternative fuel heat, such as propane, oil, or electric, and were uncertified woodstoves that stayed in place.^45^ The other 1230 or so RWH appliances were already 1988 NSPS certified and remained in the community. The Libby changeout ultimately reduced ambient air PM levels by 25%, but a follow-up study years later reported changes in the particle chemistry, as organic carbon (OC) decreased, elemental carbon (EC) remained unchanged, and emissions of resin acids increased.^46^ Later research in other U.S. rural communities (part of an asthma cohort study) discontinued the woodstove changeout intervention arm after unexpectedly finding no overall reduction in indoor PM_2.5_.^47^ Likewise, an evaluation of a woodstove changeout program in Oregon did not find a significant reduction in indoor or outdoor PM_2.5_ in participating homes.^48^ In short, the measurement of emissions and determination of current EF data for criteria pollutants, GHGs, and HAPs from older and newer U.S. wood stove technologies are critically necessary to guide environmental policies that protect air quality and public health.

Our team measured emissions of criteria air pollutants, GHGs, and HAPs from six U.S. commercially available, EPA-certified Step 2 NSPS cordwood and pellet stoves (meeting 2020 emissions targets) and one popular (circa 1980) pre-NSPS cordwood stove. We followed the ALT-140 protocol EPA is currently assessing as the next Federal Reference Method for wood stove testing,^49^ called the Integrated Duty Cycle (IDC). The IDC specifies a range for wood fuel moisture content (19 to 25%) and an operational protocol that includes multiple operational phases, fuel reloading events, various coal bed conditions, and replicate runs. This protocol, which approximates how people operate woodstoves at home, has been reported in detail previously.^50−53^ The IDC improves upon previous test protocols in multiple ways: specifying cordwood as the fuel versus structures (“cribs”) constructed from Douglas fir oven dried dimensional (2 × 4, 4 × 4) lumber; including “start-up”, high fire phase, maintenance-fire phase, and low-burn or “overnight” phase versus a single, steady state, “hot” burn condition; assessing operations with four different fuel loads with varying loading volumes and piece size rather than a single load configuration and coal bed size; and requiring a minimum of three test replicates per stove, which helps better estimate variability in reported emissions factors.^2,50^ We determined total IDC run EFs for particulate matter (PM), carbon monoxide (CO), NOx, HAPs, VOCs, metals, and GHGs, as well as other important wood combustion-generated pollutants such as non-methane total hydrocarbons (NMTHC), black carbon (as eBC), and brown carbon (BrC). Our study used two common U.S. hardwood fuel species (red maple and birch) in the cordwood stoves and commercially purchased hardwood and softwood pellets in a pellet stove. Finally, we compared RWH emissions measured in this study against the EFs used to generate emissions for the 2017 NEI (since this inventory informed EPA’s 2016 Modeling Platform). We also applied our study EFs to evaluate the national impact of RWH as a source category for key criteria pollutants and HAP risk drivers using the most recent 2020 NEI.

## 2 Materials and Methods

### 2.1 Stoves and Experimental Approach

We conducted emissions testing during 2022 and 2023 at an EPA-approved (for woodstove testing), ISO 17025 accredited laboratory (ClearStak LLC, Willington CT) on seven different appliances from four different technology categories. These included one vintage [circa 1980] pre-1988 NSPS uncertified stove, two Step 2 NSPS certified non-catalytic cordwood stoves, two Step 2 NSPS certified catalytic/hybrid cordwood stoves, and two Step 2 NSPS certified pellet stoves. Table S1 contains the detailed specifications and dimensions of the tested stoves. A U.S. EPA Level 1 Quality Assurance Project Plan (QAPP) guided testing efforts. On the basis of a previous study of Step 2 NSPS certified stoves, we chose the top performing stoves with lowest PM emissions for this research.^50^

We followed the Integrated Duty Cycle (IDC) operation and fueling protocol for cordwood and pellet stoves, except as described in Section 2.2.2 for pellet stove PAH and metals samples. The IDC provides a standard operational framework requiring a minimum of three replicate test runs as well as use of defined fueling practices, fuel sizes, moisture content range, loading densities, and cold start up and warm operation events.^50,52^ The inclusion of start-up and reloading emissions in the IDC is an important update to previous protocols used to certify wood burning appliances, which may have previously underestimated in-use PM emissions.^53^ Morin et al.^50^ described the IDC protocol in extensive detail, so we briefly summarize it here. There were four main fuel loads (or combustion phases) for cordwood stoves: startup (L1), high fire (L2), maintenance fire (L3), and overnight fire (L4). The range of moisture content of all cordwood pieces was between the IDC specified 19 and 25%. We ran the pellet stoves through eight loads (L1 to L8) of operation per the IDC protocol, with a designated startup phase (L1) and then subsequent cycling through high, medium, and low loads for 30-to-90 min intervals. Summaries of the cordwood and pellet IDC steps with explanations are provided in Table S15, panels (a) [cordwood] and (b) [pellet].

We completed six to ten IDC runs per cordwood stove technology category (uncertified, non-catalytic, and catalytic/hybrid), with a minimum of three IDC runs with maple and birch hardwood, respectively. The pellet stove combusted hardwood pellets for three IDC runs, followed by three runs with softwood pellets. ClearStak LLC locally sourced cordwood and pellets (hardwood and softwood). An external laboratory analyzed cordwood and pellet characteristics for standard parameters (i.e., percent ash, carbon, and nitrogen) and for elemental metals by ISO 16967/16968 (Table S2).

Stove flue gas flowed at an estimated 15-25 SCFM into an ASTM E2515 compliant 12″ diameter dilution tunnel. With a flow rate of ∼600 SCFM in the dilution tunnel, this results in a dilution factor range of ∼25 to 40 times the emissions from the appliance flue stack. A schematic of the dilution tunnel and the various emissions sampling locations is provided in Figure S1. Probe locations were compliant with EPA Method 1.

### 2.2 Sampling and Measurement

#### 2.2.1 Measurement of Real Time Emissions: Criteria, Greenhouse Gas, and Other Air Pollutants

Real time (1 min interval) measurements were collected in the dilution tunnel for particulate matter (Thermo Fisher Scientific model 1405-D 2-channel TEOM); carbon monoxide, methane, and formaldehyde (MKS MG2030 FTIR using EPA Method 320); THC (California Analytical HIFD-600 FID or Thermo Fisher 51-i using EPA Method 25A); NO/NO_2_/NOx (Thermo Fisher Scientific 42i Chemiluminescence, EPA Method 7E); and eBC (Magee Scientific AE22-ER or AE33 Aethalometer). The test lab sampled for equivalent black carbon (eBC) off the dilution tunnel using a Dekati eDiluter to reduce concentrations to near ambient levels. We estimated brown carbon, or BrC, as “Delta-C”,^54^ the difference between the AE33 Aethalometer reported concentrations at 370 and 880 nm (eBC) wavelengths. Figure S1 displays the sampling locations in the dilution tunnel. The TEOM operational SOP is specified in the cordwood IDC, and previous work demonstrated excellent agreement between the TEOM and filter-based PM measurements (*r*^2^ = 0.976), though TEOM values were typically 5 to 10% lower.^55,56^

All real time data were background corrected for the daily lab ambient air conditions. Emissions factors for cordwood and pellet stoves were reported on a total IDC run basis (integrated from start to finish) in units of mass of pollutant per amount of wood burned, dry basis (g/kg wood db). Non-methane total hydrocarbon (NMTHC) concentrations were determined as the difference between FID THC and FTIR methane concentrations. We estimated CO_2_ emission factors from combustion mass balance equations using the average weight fraction of carbon from test fuel characterization of wood species (Table S2) and subtracting carbon from the CO, NMTHC, and CH_4_ measurements. While we estimated CO_2_ emissions factors in this paper, we checked the validity of stove CO_2_ EFs from this work against previous work which measured CO_2_ using NDIR in-stack measurement of CO_2_ in previously performed IDC tests on the same stoves, albeit at a different test laboratory.

#### 2.2.2 Measurement of Hazardous Air Pollutants: Speciated VOCs, PAHs, and Metals

We pulled dilution tunnel gas at 2 liters/min through a stainless-steel probe and a 47 mm Tissuquartz filter (pre-baked overnight at 550 ^o^C to remove any trace organics, SKC no. 225-1823), with an attached XAD-2 adsorbent tube backup (SKC no. 226-30-06). We collected three “filter + XAD tube” samples per cordwood IDC test: during phases L1+L2 (startup and high fire loads/phases, combined), L3 (maintenance fire), and L4 (overnight fire). We shipped the samples weekly (on ice) to Enthalpy Laboratory LLC for analysis by EPA Method TO-13A for 21 PAHs using dichloromethane extraction and GC-MS analysis (Agilent Technologies Model 6890*N*/5973N Mass Selective Detector). To ensure sufficient mass for PAH analytical detection from the pellet stoves, we collected pellet PAH samples using the same sampling train described above but operated a pellet stove for continuous 4-hour periods at low, medium, and high burn settings, respectively. We calculated time-weighted total run average PAH EFs for all stoves on a mg PAH/kg wood, dry basis.

We pulled dilution tunnel gas through a different probe at 3 liters/min over 47 mm Tissuquartz filters (SKC no. 225-1823) to collect mass for metals analysis. Again, we collected three filter samples per cordwood IDC test, during phases L1+L2 (startup and high fire loads/phases, combined), L3 (maintenance fire), and L4 (overnight fire). We shipped filters to the Trace Element Analysis Core at Dartmouth College for acid digestion and analysis by inductively coupled plasma mass spectrometry (ICP-MS) for a wide suite of metals including cadmium, lead, manganese, nickel, potassium, sodium, and zinc. To ensure sufficient mass for analytical detection of metals from pellet stove operation, we collected pellet metals samples using 37 mm, 3-piece cassettes with mixed cellulose ester filters (SKC no. 225-3-01) during continuous 4-hour pellet stove operation at high, medium, and low burn settings, respectively, for both hardwood and softwood pellets. Time weighted, total run average metals EFs were calculated on a mg/kg wood, dry basis.

We sampled for speciated VOCs via collection in cleaned, evacuated Entech silonite (6L) canisters per EPA Method TO-15A off the Dekati eDiluter (Figure S2), which further diluted the tunnel concentrations with dry clean air (∼36 times) to near ambient levels (total dilution of about 1000 times). We collected three (for cordwood: L1+L2, L3, L4) or four (for pellet: L1, L2+L3+L4, L5+L6, L7) time-integrated samples per test run, along with an eDiluter dilution air blank canister per test run. We grouped pellet loads (L2+L3+L4, L5+L6) together to ensure sufficient sample was collected per canister and that pellet stove operational variability was captured (Table S15b). The Rhode Island Department of Health Laboratory performed the canister preparation and analysis for the EPA TO-15A VOC compounds using gas chromatography/mass spectrometry (Agilent GC 8890/MS 5977B). Canisters were analyzed within 7 days of sampling to minimize potential sampling artifacts.

### 2.3 Data Analysis

We performed descriptive statistics and emission calculations using Excel and Python. We reported mean, minimum, and maximum emissions factors values on a total run basis per the IDC protocol for each cordwood stove. We analyzed the pellet stove IDC run data to determine pollutant EFs for the real time measured pollutants and VOC canisters (Sections 2.2.1 and 2.2.2). As noted in Section 2.2.2, for the determination of pellet stove PAH and metals EFs, pellet stoves were run over 4 h time periods at low, medium, and high operational settings. Thus, the pellet samples (for PAHs and metals) totaled up to a maximum *n* = 24 samples [three operational settings, four (4-hour) periods, for hardwood and softwood pellets]. We averaged all the pellet EFs over all operational settings for the reported pellet stove mean EF, calculated on a pollutant mass unit (in grams or milligrams) per mass of wood fuel burned, dry basis (kilograms). All raw data were blank corrected before data analysis using the appropriate blank or background concentration per the appropriate method for each measured pollutant. To test significant differences between stove technologies for key pollutant EFs (IDC runs only), we used non-parametric one-way Kruskal Wallis ANOVA followed by Dunn’s test.

For EPA TO-15A VOC species with concentrations quantified under the minimum detection limit during a certain phase, total run VOC emissions factors followed a detailed schema to report the data set (see detailed notes in Table S3). We reported individual PAH and metals EFs when all measured concentrations per phase were above the minimum detection limit (see notes in Tables S4 and S5).

## 3 Results and Discussion

### 3.1 Real-Time Emissions: Criteria, Greenhouse Gas, and Other Air Pollutants

**Figure 1:**
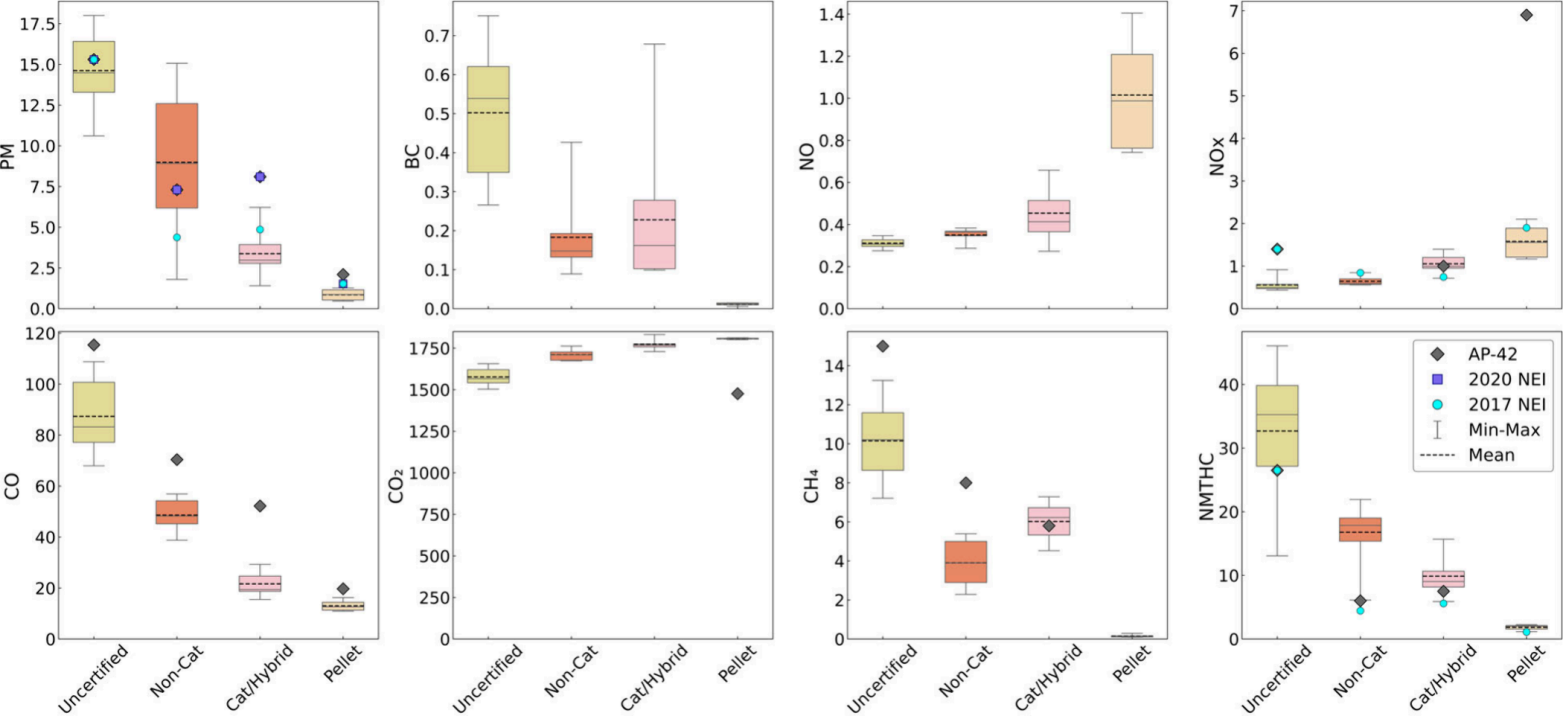
Boxplots of cumulative (total IDC run) mean emissions factors (EF, in g/kg wood burned db [on the *y*-axis]) for criteria, greenhouse gas, and other air pollutants emitted by uncertified (*n* = 10 IDC runs), non-catalytic (*n* = 9), catalytic/hybrid (*n* = 9), and pellet stoves (*n* = 6), respectively. We pooled the data for cordwood stoves (maple and birch) and pellet stoves (hardwood and softwood). Compounds were measured in real-time (1 min interval) and integrated over the entire IDC run. The various sampling methods are explained in Section 2.2.1. The boxplots represent the entire range of data, mean is the dotted line, median is the solid line, and the whiskers are the minimum to maximum EF value. Each stove technology is also compared to EFs from EPA AP-42 (grey ◆), the 2017 NEI (blue ●) and the 2020 NEI (purple ■).

Table 1 and Figure 1 summarizes key emissions factors per stove technology type (pooled for maple and birch, and for hardwood and softwood pellets) for multiple criteria, greenhouse gas, and related air pollutants. The uncertified cordwood stove PM EF (14.6 g/kg) was 1.6 times higher than the non-catalytic EF (8.98 g/kg), 4.3 times higher than the catalytic stove EF (3.38 g/kg), and 17.0 times higher than the pellet EF (0.86 g/kg). Uncertified stoves emitted the highest PM of all stove technologies, followed by non-catalytic, catalytic/hybrid, and pellet categories, respectively. The pellet stove PM EF was significantly lower than all cordwood stove PM EFs, except catalytic/hybrid (*p* < 0.01) (Table S10). Interestingly, there was no significant difference between uncertified and non-catalytic stove PM EFs. This result may have implications for the benefits of non-catalytic stoves in woodstove changeout interventions, as an indoor air quality study of a woodstove exchange program also did not find a significant PM reduction benefit from non-catalytic stoves.^48^ However, as the non-catalytic category had the highest variability in the data, and only two models were tested in this study, additional research evaluating multiple U.S. non-catalytic stoves is needed.

**Table 1:** Cumulative Mean Emissions Factors (EF, g/kg Wood, Dry Basis) Plus the EF Range [Min, Max] for PM, BC, BrC, NO, NOx, CO, CO_2_, THC, NMTHC, CH_4_, and Formaldehyde (CH_2_O) Determined Following the IDC Test Protocol, for *n* = 6 to 12 Runs Per Stove Category^a^

	Emissions Factors (g/kg): Mean [min,max]*_n_*			
Species	Uncertified	Non-Cat	Cat/Hybrid	Pellet
PM	14.6 [10.6, 18.0]_10_	8.98 [1.79, 15.07]_12_	3.38 [1.41, 6.22]_9_	0.86 [0.46, 1.27]_6_
Black carbon (eBC)	0.50 [0.27, 0.75]_10_	0.18 [0.09, 0.43]_9_	0.23 [0.10, 0.68]_9_	0.012 [0.006, 0.015]_6_
Brown carbon (BrC)	1.85 [1.49, 2.36]_7_	0.63 [0.25, 1.01]_6_	0.75 [0.43, 1.83]_6_	–
NO	0.31 [0.28, 0.35]_10_	0.35 [0.29, 0.38]_9_	0.45 [0.27, 0.66]_9_	1.02 [0.74, 1.40]_6_
NOx	0.56 [0.44, 0.92]_10_	0.64 [0.56, 0.85]_9_	1.05 [0.71, 1.40]_9_	1.58 [1.16, 2.10]_6_
NO_2_	0.10 [0.02, 0.39]_10_	0.12 [0.02, 0.30]_9_	0.36 [0.21, 0.48]_9_	0.032 [0.000, 0.057]_6_
CO	87.4 [68.0, 108.8]_10_	48.6 [38.8, 56.9]_9_	21.7 [15.5, 29.3]_9_	13.0 [10.9, 16.3]_6_
CO_2_	1577 [1503, 1656]_10_	1711 [1673, 1762]_9_	1774 [1729, 1832]_9_	1807 [1801, 1812]_6_
CH_4_	10.15 [7.21, 13.24]_10_	3.90 [2.28, 5.39]_9_	6.02 [4.52, 7.29]_9_	0.15 [0.09, 0.29]_6_
CH_2_O	2.14 [1.74, 2.43]_10_	1.16 [0.75, 1.47]_9_	1.08 [0.70, 1.41]_9_	0.25 [0.21, 0.31]_6_^b^
THC	42.7 [21.6, 58.0]_10_	20.6 [8.4, 27.2]_9_	15.8 [12.3, 21.3]_9_	1.98 [1.27, 2.41]_6_
NMTHC	32.7 [13.1, 46.1]_10_	16.8 [6.1, 21.9]_9_	9.88 [5.88, 15.69]_9_	1.83 [1.15, 2.25]_6_

a We pooled the data for cordwood stoves (maple and birch) and pellet stoves (hardwood and softwood). −, means not detected; the number of IDC runs, *n*, is subscripted next to the brackets.

b < MDL.

The maple versus birch fuel species did not influence cordwood PM EFs, except in the non-catalytic category, with the average birch PM EF almost 2 times higher than the average maple PM EF (Figure S3A). The elevated PM from birch may be related to different combustion characteristics of the fuel, including bark that tends to remain on birch logs compared to other wood species. Kortelainen et al. performed a detailed study of time resolved PM and BC emissions for birch, beech, and spruce, determining the highest PM and BC emissions from birch (both 1.4 times higher than beech hardwood).^57^ There were also multiple BC peaks per fuel load, attributed to birch bark on the logs.^57^ Higher PAHs from birch may also influence PM formation.^58^ We recommend additional research into the influence of the combustion phase on PM emissions as well as increasing the sample size of birch fuel. Pellet stoves using hardwood fuel emitted 2.2 times the PM as softwood pellets (Figure S3A). The range (min, max) of RWH PM emissions was highly variable, even within the same stove technology category and with multiple replicate runs.

While RWH appliance technologies, fuel species, moisture content, testing methods, and operational protocols differ markedly across research studies globally, we do note general patterns in comparing our work to others. PM EF results from the uncertified and noncatalytic stoves in this study far exceeded EFs measured by others^59,21^ but were comparable to some studies^30,60^ (see Table S11). Comparing this study to previous NESCAUM IDC experiments on different stove models but similar technology,^50^ the non-catalytic PM EFs were slightly elevated (1.1 to 1.3 times higher) and the pellet PM EFs were within the previous range observed (0.5 g/kg to 2.0 g/kg). Evaluating earlier NESCAUM research and our results here suggest the importance of both the operational protocol and using a range for cordwood moisture content on PM EF results. In the studies we reviewed, many from Europe (Table S11), the wood moisture content was in the 10 to 18% range, whereas most U.S. homeowners’ cordwood moisture content levels in wood are likely around 20 to 25%, if not higher.^52^ Higher PM EFs were observed in almost all cases when moisture content in cordwood increased from 21% to 27–29%.^52^ Using NESCAUM PM EFs in the 2020 EPA NEI determined total PM emissions of 21,100 tons from catalytic stoves in the U.S., 2.4 times lower than the 2020 NEI estimated 50,600 tons (Figure S16). Conversely, PM emissions from non-catalytic stoves were roughly 82,400 tons, or about 1.2 times higher compared to the 2020 NEI output of 67,000 tons (Figure S16).

In contrast to PM, NOx EFs were less variable but increased with newer stove technology, with pellet stoves emitting the most NOx (pellet > catalytic/hybrid > non-catalytic > uncertified). Hardwood pellets emitted 1.6 times more NOx than softwood (1.94 vs 1.22 g/kg, respectively). This is likely due to higher combustion temperatures. Applying the NESCAUM EF shows NOx emissions are overestimated in the 2020 NEI for all stove technologies except catalytic/hybrid (Figure S18). Carbon monoxide EFs followed the same trend as PM where uncertified > non-catalytic > catalytic/hybrid > pellet stove EFs. The pellet CO EF was significantly lower than the cordwood stove EFs (*p* < 0.01), except for catalytic/hybrid versus pellet. CO EFs did not exceed the AP-42. Comparing our NOx and CO EFs to other studies, the average EF results from our study were up to 2 times lower than EFs reported by others, depending on the stove type (Table S11).^30,60^

The NMTHC EF trend (uncertified > non-catalytic > catalytic/hybrid > pellet stove) was similar to PM and CO, except all stoves including pellet exceeded the AP-42, 2017 and/or 2020 NEI EFs (Figure 1). There was no significant difference in NMTHC EFs between uncertified and noncatalytic categories, though pellet was significantly lower in comparison (*p* < 0.01 and *p* < 0.05, respectively). The 2020 NEI uses AP-42 EFs (for NMTHC) to determine total VOC emissions from RWH. When we applied the NMTHC EFs from this study (Figure S19), we quantified total VOC emissions from cordwood and pellets stoves at roughly 356,000 tons per year compared to the 2020 NEI estimate of about 215,000 tons per year. This result is highly influenced by the non-catalytic stove category, where VOC emissions are approximately 2.8 times higher than the current 2020 NEI EF for this technology category (Figure S19).

RWH appliances, especially uncertified cordwood stoves, were a notable source of methane (CH_4_), which is a potent GHG (Table 1 and Figure 1). Uncertified stoves emitted significantly higher CH_4_ compared to all other stove types (*p* < 0.01, pellet and noncatalytic, *p* < 0.05, catalytic/hybrid). Others^60,23^ have reported cordwood stove CH_4_ EFs similar to this study. Emissions of CH_4_ from pellet stoves were the lowest of any stove technology tested, but not trivial (Table 1). Importantly, even the pellet stove CH_4_ EF was approximately 7× higher than CH_4_ emissions from natural gas heating and 5× higher than oil heating.^61,62^ We recommend the inclusion of CH_4_ emissions from RWH when assessing greenhouse gas emissions from residential heating.

Carbon dioxide EFs followed the trend pellet > catalytic/hybrid > non-catalytic > uncertified, with the pellet CO_2_ EF exceeding the AP-42. The pellet stove CO_2_ EF was significantly higher than uncertified (*p* < 0.01) and noncatalytic (*p* < 0.05) but not catalytic/hybrid. Though pellet fuel CO_2_ EFs are approximately 3× lower compared to residential oil heat CO_2_ EFs, and the wood growth cycle may offset CO_2_ emissions further,^29^ RWH as a category still emits notable CO_2_ emissions. For BC, considered a short-term climate forcer,^63^ this study’s non-catalytic stove eBC EF was comparable to BC EFs reported by others.^21−23^ Pellet stoves emitted significantly less eBC compared to uncertified (*p* < 0.01) and noncatalytic and catalytic/hybrid stoves (*p* < 0.05). The non-catalytic eBC EF was slightly lower than the catalytic/hybrid, but this difference was not significant.

Brown organic carbon, or BrC, was determined as “Delta C”, the difference in 370 and 880 nm wavelengths in the Aethalometer. “Delta-C” is a semi-quantitative measure of BrC, which is considered important for its role in UV wavelength absorbance and contribution to atmospheric warming.^36,37^ The uncertified stove BrC EF was 3 times higher compared to the non-catalytic stove BrC EF and 2.5 times higher than the catalytic/hybrid category, which was strongly influenced by maple cordwood (see Table 1 and Figure S3B). This is somewhat in contrast to a detailed study of BrC contributions from RWH in Europe, where BrC was influenced by fuel moisture content, combustion efficiency, and loading phases but deemed less influenced by stove technology or wood fuel species.^36^ Thus, additional research on U.S. stoves’ BC and BrC emissions are needed, as evidence suggests RWH BrC contributions from rural areas in Europe may be underestimated,^37^ along with the climate forcing capacity from local RWH aerosols. Comparing RWH against other heating fuels, while oil heating appliances emit far more CO_2_,^29^ the comparatively high CH_4_ and BC EFs from RWH raise concerns regarding the impact of RWH as a climate mitigation strategy to reduce warming. Multiple researchers have evaluated non-CO_2_ emissions from RWH, including CH_4_, CO, BC, and NMTHC, concluding these short-term climate forcers contribute to overall increased warming, offsetting the CO_2_ emissions reduction benefits.^63−65^

### 3.2 Measurement of Hazardous Air Pollutants: Speciated VOCs, PAHs, and Metals

**Figure 2:**
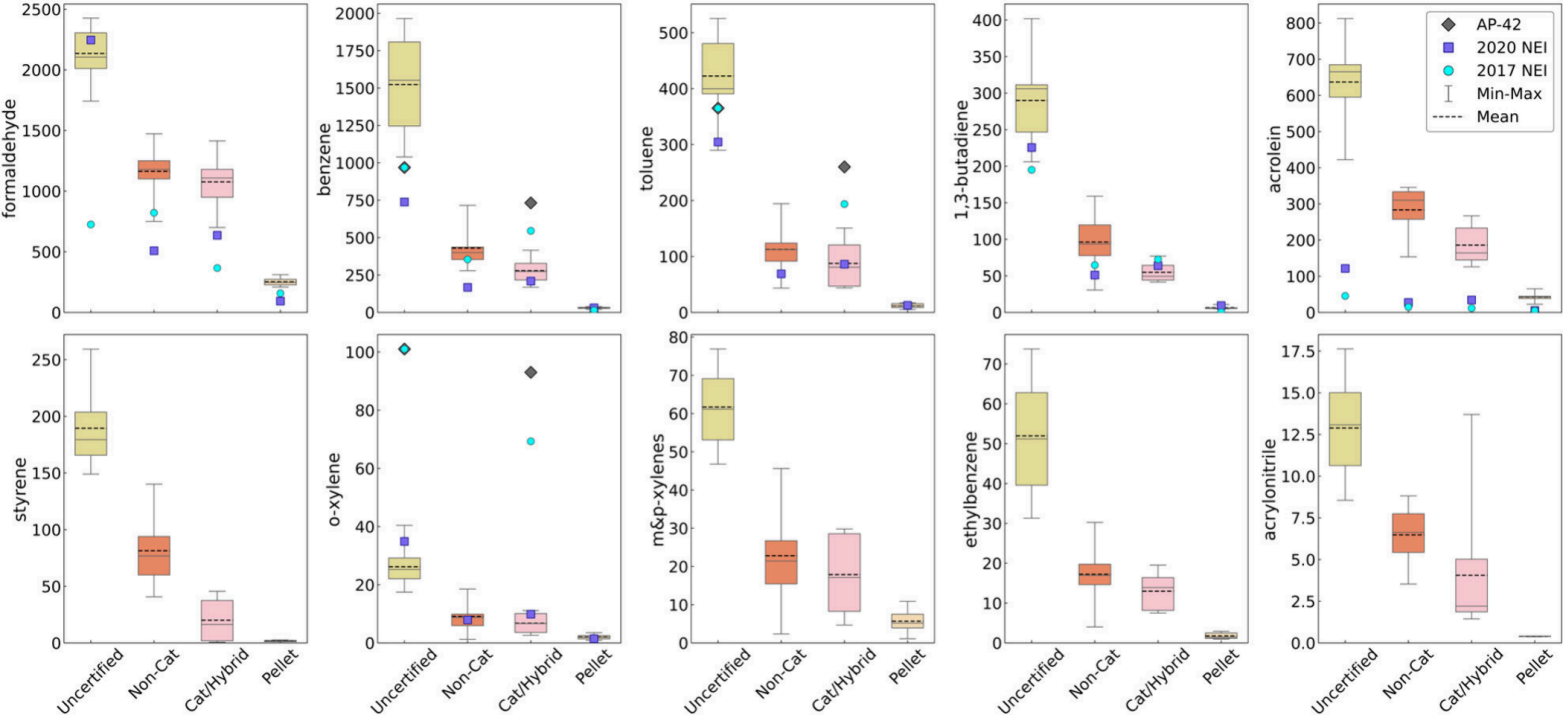
Cumulative mean emissions factors (EF, in mg/kg wood burned db) for speciated volatile organic compounds (VOCs) emitted by uncertified (*n* = 10 IDC runs), non-catalytic (*n* = 9), catalytic hybrid (*n* = 9), and pellet stoves (*n* = 6), respectively, following the IDC protocol for each experimental run. To determine the total number of samples (canisters) per VOC, see Table S3. We pooled the data for cordwood stoves (maple and birch) and pellet stoves (hardwood and softwood). The boxplots represent the entire range of data, mean is the dotted line, median is the solid line, and the whiskers are the minimum to maximum EF value. Compounds were measured by EPA Method TO-15A except for formaldehyde, which was measured by EPA Method 320. We compare each stove technology EF from this study to EFs from EPA AP-42 (where applicable, grey ◆), and the NEI from 2017 (blue ●) and 2020 (purple ■). All compounds in this plot are on EPA’s list of HAPs. Additional speciated VOC EFs and number of IDC runs are listed in Tables S3 and S7, and statistically significant differences are listed in Table S10.

As seen in Figure 2, we found that RWH cordwood appliance EFs for speciated VOCs followed the general trend of uncertified > non-catalytic ≥ catalytic/hybrid ≫ pellet (Table S3). Formaldehyde (Table 1) as a single VOC was emitted in the range from 1 to 2.1 g/kg, and benzene (Table S3) was in the range from 0.3 to 1.5 g/kg from cordwood stoves. Comparing these individual VOC emissions to oil heat, residential oil burners contribute total VOC emissions in the range from 6 to 30 mg/kg of fuel burned.^66^ Formaldehyde, benzene, toluene, 1,3-butadiene, acrolein, and styrene had the highest EFs across all cordwood stove types in this study. Uncertified stove speciated VOC EFs were significantly higher than non-catalytic stove EFs (*p* < 0.05) except for benzene, 1,3-butadiene, acrolein, pentane, and styrene (Table S10). There were no significant differences observed between the non-catalytic and catalytic/hybrid stove category for any individual VOC EF. Only the pellet stove category consistently emitted significantly less individual VOCs compared to the uncertified stove (*p* < 0.01). Use of non-catalytic or catalytic/hybrid stoves resulted in a notable 2-to-3-fold reduction in multiple individual VOC EFs compared to uncertified technology, with pellet stove EFs offering orders of magnitude reduction (Figure 2, Table S3). In comparison to NEI EFs, the non-catalytic stove category individual VOC EFs ranged from 1.2 to 19 times higher than the NEI 2017 or NEI 2020 regulatory EFs for formaldehyde, benzene, toluene, 1,3-butadiene, and acrolein (Figure 2).

While the NEI includes the HAPs formaldehyde, benzene, toluene, 1,3-butadiene, and acrolein, AP-42 provides no data for these emissions. When we applied this study’s EFs to determine these pollutants’ emissions from all RWH appliances in the 2020 NEI, RWH ranked as the second largest source of formaldehyde, a compound considered by the EPA National Air Toxics Assessment (NATA) as a national cancer risk driver (Figure S10). RWH ranked as the third largest source of emissions for acrolein, benzene, and 1,3-butadiene in the 2020 NEI, with the latter two compounds considered NATA national cancer risk contributors. Figures S10–S14 show the tons per year emitted and the relationship of RWH to other sources of these HAPs, such as all fires (wildfires, prescribed burns, and agricultural burning combined) and mobile sources (all road and nonroad sources, combined). To estimate the total tons per year of these individual HAPs, we assessed the full universe of RWH appliances in the 2020 NEI Residential Wood Combustion (RWC) category. First, we applied the speciated VOC EFs per stove category from this study, and we next applied the uncertified stove speciated VOC EF to other uncertified appliances in the RWC source category (i.e., hydronic heaters, furnaces, fireplaces, and outdoor wood boilers) and applied the non-catalytic stove EF to certified RWH appliances (Table S3). For pellet RWH appliances, the pellet EF was applied.

With regard to other HAPs, cordwood stoves emitted elevated quantities of acrylonitrile, ethylene oxide, styrene, ethylbenzene and m- and p-xylenes (Table S3). The uncertified stove emitted significantly higher ethylene oxide, toluene, o-xylene, m- and p-xylene, and ethylbenzene compared to all other stove technologies (*p* < 0.05). We did not observe an effect from wood fuel species (maple versus birch) on gaseous VOC emissions. We also measured low, < MDL, or undetected chlorinated VOCs by EPA TO-15A from the RWH appliances tested in this study, an important result for this HAP category (see Tables S3 and S7). Comparing the average formaldehyde EF from our non-catalytic stoves (1160 mg/kg) to other emissions studies of non-catalytic stoves in the literature (many using European tree species), our average EF was 1.5 times lower than 1775 mg/kg from Pyrenean oak wood;^67^ comparable to 1290 mg/kg from Canadian oak;^60^ but ∼2 to 4.7 times higher than the average EFs of 590 mg/kg from beech wood,^23^ 350 mg/kg from spruce,^59^ and 246 mg/kg from oak.^30^ For a complete list of the quantified VOC EFs from this study see Tables S3 and S7. To evaluate those VOC EFs that significantly differed by technology, see Table S10. Finally, for a more detailed comparison of this study’s VOC EFs to values in the literature, see Table S13.

**Figure 3:**
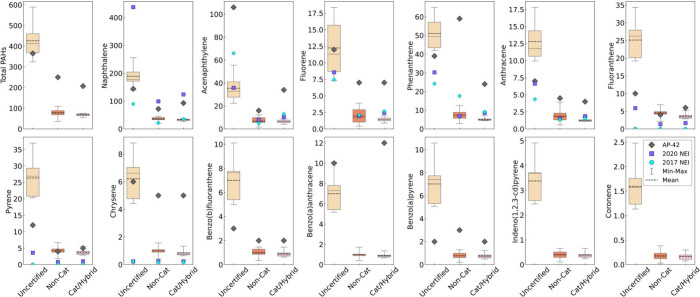
Cumulative mean emissions factors (EF, in mg/kg wood burned db) for PAHs emitted by uncertified (*n* = 7 IDC runs), non-catalytic (*n* = 9), and catalytic hybrid stoves (*n* = 6), respectively, following the IDC protocol for each experimental run. To determine the total number of samples per PAH, see Table S4. We pooled the data for cordwood stoves (maple and birch). The boxplots represent the entire range of data, mean is the dotted line, median is the solid line, and the whiskers are the minimum to maximum EF value. Compounds were measured by EPA Method TO-13A. Each stove technology is also compared to EFs from EPA AP-42 (grey ◆), and the EPA NEI from 2017 (blue ●) and 2020 (purple ■). Additional PAH EFs and number of IDC runs are listed in Tables S4 and S8. Statistically significant differences are listed in Table S10.

Figure 3 and Tables S4 and S8 show RWH PAH emissions, with uncertified stoves > non-catalytic ≈ catalytic/hybrid. We observed only naphthalene, acenaphthylene, 2-methylnapthalene, and 1-methylnapthalene in pellet stove emissions, with pellet EFs ∼ 1 mg/kg. Since no other PAHs were detected, pellet stove emissions were not found to be a notable source of PAHs and were not included in Figure 3. Uncertified stoves emitted significantly higher PAHs for all species in Figure 3 including naphthalene (a NATA national risk contributor) and total PAHs, compared to non-catalytic and catalytic/hybrid stoves (*p* < 0.05, Table S10). Non-catalytic and catalytic/hybrid PAH EFs were quantitatively similar (no significant differences) and approximately 10 times lower than uncertified PAH EFs, showing an immediate PAH reduction from these Step 2 cordwood stoves. The uncertified stove EFs for most PAHs in Figure 3 were up to 2.5 times higher than AP-42 EFs and up to 5 times the NEI 2020 EF. The uncertified stove EFs for fluoranthene and pyrene exceeded the NEI 2017 EF by orders of magnitude.

We also quantified particle-phase PAHs such as benzo(a)pyrene, indeno (1,2,3-cd) pyrene, and coronene from all cordwood stove types. Compared to other studies in the literature, our non-catalytic PAH EFs were an order of magnitude higher than most individual PAH EFs reported by Bruns et al. (2015),^21^ though the latter study used a denuder before the quartz filter and had far lower PM emissions (0.3 g/kg) than our study’s 8.98 g/kg (Table S12). Our non-catalytic stoves’ individual PAH EFs were comparable to results determined by McDonald et al., which also captured more volatile PAHs like naphthalene with their sampling train.^30^ We also determined EFs for particle-phase PAHs such as benzo(a)pyrene and indeno(1,2,3-cd)perylene similar to levels reported in a study, which measured particle-phase PAH on aluminum filters.^31^ Notably there are few studies within the last 20 years analyzing PAH EFs from uncertified stoves (Table S12), with ECCC^60^ reporting total PAH EF at 118 mg/kg (no individual species reported at this time), a value 3.6 times lower than determined in our study. However, as noted earlier, the differences between burn protocols, stove type, wood fuel species, moisture content, as well as the inherent variability of RWH appliance operation suggests that a range of values may be a more appropriate representation for cordwood stove PAH EFs. Additionally, PAH sampling methods in the literature vary in their capture and identification of semi-volatiles (due to complex gas-phase and particle-phase partitioning dynamics). We suggest additional research efforts to quantify PAH emissions from U.S. RWH technologies to build a robust database for emissions inventories.

**Figure 4:**
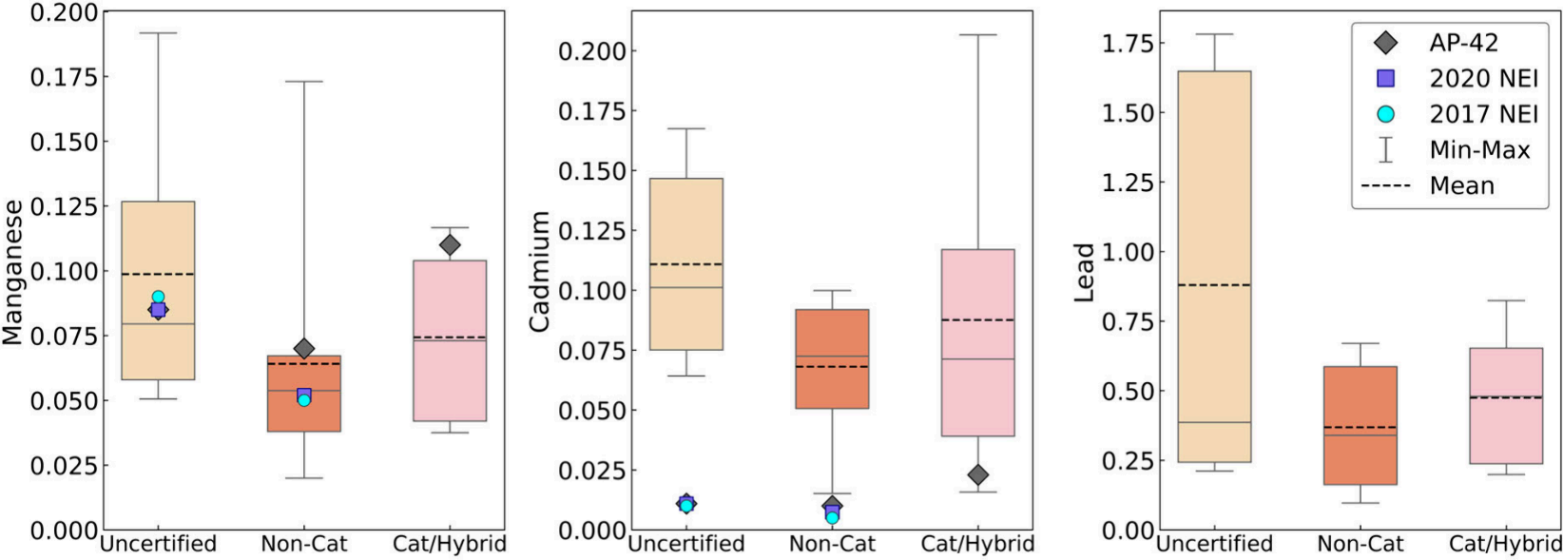
Total stove run cumulative mean emissions factors (EF, in mg/kg wood burned db) for toxic metals emitted by uncertified (*n* = 7 IDC runs), non-catalytic (*n* = 6), and catalytic hybrid cordwood stoves (*n* = 6), respectively, following the IDC protocol for each experimental run (see Table S5 for the number of samples per metal). We pooled the data for cordwood stoves (maple and birch). Pellet stoves (hardwood and softwood) are reported in Figure S8. The boxplots represent the entire range of data, mean is the dotted line, median is the solid line, and the whiskers are the minimum to maximum EF value. Each stove technology is also compared to EFs from EPA AP-42 (grey ◆) and the NEI from 2017 (blue ●) and 2020 (purple ■). Additional EM EFs are listed in Table S5. Statistically significant differences are listed in Table S10.

Figure 4 displays the results of toxic metals emitted from cordwood stoves in this study, with additional results in Tables S5 and S9. Figure 4 does not display the pellet stove metals EFs, as the pellet stove was not tested per the IDC protocol but rather tested during multiple 4-hour time periods at different operational settings (see sections 2.2.2 and 2.3, and for the pellet results, see Table S5 and Figure S8). Mean Mn and Cd EFs from uncertified and non-catalytic stoves exceeded 2017 and 2020 NEI EFs, and cordwood Pb EFs were higher than all other toxic metal EFs (Figure 4 and Table S5). Exposure to these metals is of high human health concern according to the EPA IRIS database, and these metals have been measured in wood heat indoor air quality studies.^25,68,69^ Currently, the AP-42 or NEI do not consider RWH as a source of Pb, though Pb emissions from wildfires were recently included in the 2020 NEI. If we apply the Pb EF from this study to all RWH (including residential cordwood boilers, hydronic heaters, fireplaces, and furnaces by applying the uncertified Pb EFs to uncertified appliances, the noncatalytic Pb EF for certified appliances, and pellet Pb EF for pellet appliances), RWH would be the 4th largest source of Pb in the NEI at 50,112 pounds, above wildfires (Figure S9). While Pb is a criteria air pollutant more commonly associated with aviation gasoline fuel and the historic use of leaded gasoline in cars, our results indicate that RWH may be an underestimated source of Pb in ambient air to consider in future emissions inventories, particularly for impact to disadvantaged and EJ communities.

There were no significant differences in Pb, Mn, and Cd EFs between cordwood technology types. Interestingly, wood fuel species type influenced Pb, Cd, and Mn emissions from uncertified and catalytic/hybrid stoves, with birch hardwood emitting more than maple (Figures S4–S6), but there was no such influence in the non-catalytic category. Referring to Table S2, the fuel characterization analysis of the cordwood itself showed four times higher concentration of Pb and two times higher concentration of Cd in birch cordwood compared to maple. Similar analysis of the hardwood versus softwood pellets showed higher Mn, Cd, and Pb in hardwood pellets (Table S2). Previous studies of pellet composition have also found elevated levels of Pb, Mn, and Cd in multiple commercial pellet samples.^70,71^ Researchers of forest soil throughout the Northeast, where our fuel stock originated, have continued to find Pb content in forest soils due to historic deposition of lead from gasoline combustion.^72,73^ Forest floor samples taken from New York’s Adirondacks, western Connecticut, and western Massachusetts contained Pb concentrations of approximately 90 mg/kg.^72^ A recent study reported Pb EFs from laboratory biomass combustion of forest litter ranging from 0.064 mg/kg, 0.125 mg/kg, and 0.526 mg/kg from sites in North Carolina, Montana, and Minnesota, respectively.^74^ Finally, researchers have identified wildfire smoke from intense fire events in California as a potential source of Pb in ambient air.^75^

As seen in Table S14, others have reported elevated Pb and Mn from cordwood combustion in European stoves, with 12.2 mg/kg (Pb) and 11.6 mg/kg (Mn) reported for Portuguese oak,^76^ Pb at 1.20 mg/kg for spruce,^59^ and 0.16 mg/kg (Pb) and 0.14 mg/kg (Mn) reported burning birch.^33^ Since both Pb and Mn are easily taken up by plants, biomass burning may volatilize these trace metals.^76^ In another study, stove EFs were reported under the minimum detection level for Mn but 0.04 mg/kg Pb in no-catalyst mode and 0.11 mg/kg in catalyst mode from oak combustion in a U.S. catalytic cordwood stove.^32^ Interestingly, in our study, for pellet combustion, Cd and Pb emissions were 2.4 and 5.6 times lower when fueled by softwood pellets compared to hardwood; Mn EFs were essentially the same (0.33 mg/kg [hardwood] vs 0.32 mg/kg [softwood]). Beyond Mn, Cd, and Pb, our study also determined As and Ni in cordwood and pellet stove emissions (Tables S5 and S9). Pellet stove EFs for Mn and As were also approximately 3 to 10 times higher than cordwood stoves. While the pellet operational protocol was different, precluding statistical testing, our results suggest pellet stoves’ toxic metals EFs may be similar to, and for some toxic metals greater than, cordwood EFs (Figure S8). We recommend additional research into RWH as a source of toxic metals in ambient air in the U.S., especially in rural areas throughout the country.

A recent EPA National Air Toxics Assessment examined co-variance patterns of HAPs and concluded that at rural monitoring sites, PAHs were found as one cluster; and *p*-dichlorobenzene and speciated heavy metals such as Ni, Be, and Cd were found as another cluster.^24^ Because RWH is a major source of PAHs in rural areas, we created criteria, HAP and GHG pollutant heatmaps for each of the cordwood technologies to explore apparent trends or patterns (Figures S15a–c). As expected, individual PAHs in each stove technology heatmap were highly correlated with each other. Metals were also positively correlated with each other, except for Ni in catalytic/hybrid stoves. This unexpected Ni result may be due to a comparatively small sample size for Ni or other reasons related to the catalytic technology itself. PM in noncatalytic stoves was highly positively correlated with CO, CH_4_, and NMTHC. PM correlations with these and other pollutants were weaker when comparing across the other technology types. In fact, PM is not strongly positively correlated with any other pollutant in the catalytic stove technology, though PM is negatively correlated with CO_2_ and total PAHs, which may be due to higher temperatures and improved combustion. Overall, the heat maps in Figure S15 differed notably between the cordwood stove technologies, particularly for PM, VOCs, eBC, and certain PAHs and metals. We hypothesize this may be due to the impact of the various combustion technologies and phase to phase burn conditions, which calls for future research into these factors on stove technology emissions profiles.

While we recommend more RWH research in laboratory and field settings to further improve emissions inventories and understanding of RWH impacts on public health and climate, deploying a similarly comprehensive sampling and analysis plan requires a substantial commitment of resources, especially in the measurement of HAPs in the field. With that perspective, we investigated the relationship of NMTHC (determined by “EPA Method 25A THC” minus “EPA Method 320 CH_4_”) with individual HAP VOCs as determined by EPA TO-15A. We plotted total run NMTHC emission rates from all cordwood stove types versus the total run emission rates of individual HAP VOCs including benzene, toluene, styrene, acrolein, and others (Figure S4). The linear regressions were generally in good agreement, with *r*^2^ values that ranged from 0.68 to 0.80, depending on the specific VOC. This shows promise for use of real-time instrument technology such as flame ionization detection to help estimate concentrations of key HAP VOCs from RWH in “real world” studies, though more research in this area is needed.

The health impacts from wood smoke exposure are a serious public health issue, especially for those living in rural, low-income, and disadvantaged communities.^2,40,77^ Emissions factors are a critical data input into federal, state, local, and tribal data analysis, underpinning air toxics programs, climate action plans, state implementation plans, and overall air quality decision-making to protect public health at the national, state, local and tribal levels. Applying EFs from this study into the 2020 NEI determined that RWH is in the top 4 largest source categories for Pb (Figure S9) formaldehyde (Figure S10), 1,3-butadiene (Figure S11), acrolein (Figure S12), and benzene (Figure S13). We determined that RWH EFs for other HAPs, PAHs, methane, Mn, Cd, and BC were also notable compared to other researchers’ results (Table S11–S14). We did not find any statistically significant differences in toxic metals EFs (Mn, Cd, Pb, As) between the cordwood stove technologies. Additionally, pellet stove metals EFs were quantitatively similar to cordwood EFs, but because the pellet sampling protocol for metals did not follow the IDC, we could not apply statistical tests to the pellet vs cordwood metals comparisons. We recommend more research into RWH as a source of VOC HAPs and heavy metals in ambient air, as this is an understudied area with public health impacts for rural and EJ communities that use wood heat.

This study found that contrary to EPA RWH emission factors, catalytic stoves emit lower emissions for PM and CO than non-catalytic stoves. Unexpectedly, uncertified stove EFs were not significantly different from Step 2 non-catalytic EFs for PM, CO, NMTHC, metals, and multiple HAPs including benzene, 1,3-butadiene, acrolein, pentane, and styrene. The pellet stove category was the only consistent technology to significantly reduce most criteria, eBC, and HAP emissions compared to the noncatalytic stoves (*p* < 0.05), except for NOx, CH_4_, pentane, metals, o-xylene, and m- and p-xylene.

We acknowledge the limitations of this study. On the basis of previous work,^50^ we evaluated two lower-PM-emitting stove models each in the non-catalytic and catalytic/hybrid technology categories, two pellet models and one uncertified model, for a total of seven stoves. Future research should expand to include other U.S. stove models in the non-catalytic, catalytic, and pellet categories, especially popular retail models from different areas of the country. We did not measure HAPs that others have noted are emitted by RWH in substantial quantities, such as alcohols, carbonyls, phenols, furans, oxygen-containing organic carbon species, and other compounds.^23,30,32,78,79^ Furthermore, understanding the contribution of RWH HAPs and VOCs to primary organic aerosol (POA) and VOC ambient air concentrations is important to assess secondary organic aerosol (SOA) formation processes, particularly as combustion technologies evolve.^21,23^ While we did not explicitly measure the organic carbon (OC) fraction of aerosols or POA in this study, the impact of RWH on SOA formation processes is a critical area for future research. The operational protocol also has an impact on the generation of SOA, PAHs, and other VOCs. Bruns et al.^21^ reported that “high” fuel loadings shifted the contribution of PAHs to approximately 15% of the total organic aerosol, compared to only 4% from “average” fuel loading. These researchers further identified important SOA precursors in woodsmoke, highlighting benzene, alkyl-benzenes, phenols, naphthalene, and alkyl-naphthalene, many of which are also HAPs that impact human health (Bruns et al.).^80^

Taken as a whole, the data suggest complex trade-offs for policy-makers attempting to balance climate mitigation and public health goals using cordwood technologies. EPA Step 2 certified non-catalytic appliances may not have the intended benefit of improving overall air quality if a substantial percentage of the stoves exchanged in woodstove changeout programs use this control technology. While the catalytic/hybrid and pellet stoves in this study had the lowest PM and PAH emissions, methane emissions were not significantly lower compared to the non-catalytic category, and metals emissions appear quantitatively similar between all stove technologies. Additional research on PM, HAPs, and GHGs is recommended, especially as the new PM NAAQS will likely classify new non-attainment areas due primarily to emissions from RWH. Finally, due to current development of GHG reduction strategies at the local to global level, we suggest further evaluation of RWH GHG/climate forcing emissions (CO_2_, CH_4_, BC, BrC) compared to more conventional heating fuels, such as oil or natural gas, as well as new heating technologies. Many current GHG models for residential heating do not include RWH, potentially overlooking CH_4_, BC, and BrC emissions from this source and raising questions on the climate benefit from wood heating. Future research should also examine the impact of different burn or phase conditions during standard testing protocols such as the IDC on criteria and GHG and HAPs emissions from common commercial RWH technologies in the U.S.

## Supporting Information Available

Stove specifications (Table S1), test fuel characterization (Table S2), emissions factors for all compounds measured in the study (in units of mass/kg wood burned and mass/unit of stove output energy (MJ) (Tables S3 to S9), ANOVA comparisons (Table S10), comparisons of this study’s emissions factors to other studies in the literature (Tables S11–S14), and summaries of the Integrated Duty Cycle test method for cordwood and pellet stoves (Table 15a,b). We also include figures of the measurement set-up (Figure S1), the 2020 EPA NEI PM emissions by source category (Figure S2), various emissions by wood fuel species (Figure S3a,b, Figures S5–S7), pellet stove metals emissions (Figure S8), regressions and heat maps comparing compounds or compound categories (Figures S4, S15a–c). Finally, we applied this study’s emissions factors in the 2020 EPA National Emissions Inventory to show the potential impact of Residential Wood Combustion as a source category, nationally (Figures S9–S14), further comparing EFs by technology category and NEI year (Figures S16–S19) (PDF)

## Abbreviations

RWH: residential wood heating
RWC: residential wood combustion
EPA: Environmental Protection Agency
EF: emissions factor
NEI: National Emissions Inventory
PM: particulate matter
eBC: equivalent black carbon
BrC: brown carbon
CO: carbon monoxide
CO_2_: carbon dioxide
NOx: nitrogen oxides
CH_4_: methane
GHG: greenhouse gas emissions
PAH: polycyclic aromatic hydrocarbon
VOC: volatile organic compound
HAP: hazardous air pollutant
IDC: integrated duty cycle
eBC: equivalent black carbon
THC: total hydrocarbons
NMTHC: non methane total hydrocarbons
NSPS: new source performance standards
QAPP: quality assurance project plan
TEOM: tapered element oscillating microbalance
FTIR: Fourier transform infrared
SOA: secondary organic aerosol
