# Supplementary material for: Criteria, Greenhouse Gas, and Hazardous Air Pollutant Emissions Factors from Residential Cordwood and Pellet Stoves Using an Integrated Duty Cycle Test Protocol
Source: ACS EST Air. 2024 Aug 12;1(9):1190–202. doi: 10.1021/acsestair.4c00135 (PMC11406481; doi:10.1021/acsestair.4c00135)
Supplement: Supplementary file 1 — ea4c00135_si_001.pdf [file ea4c00135_si_001.pdf]

Supporting Information

Title: *Criteria, greenhouse gas, and hazardous air pollutant emissions factors from residential cordwood and pellet stoves using an integrated duty cycle test protocol*

Authors: Nora Traviss*[1,2], George Allen[1], Mahdi Ahmadi[1,3]

*ntraviss@nescaum.org, corresponding author

[1] Northeast States for Coordinated Air Use Management (NESCAUM), Boston, MA, 02111, USA

[2] Keene State College, Keene, NH, 03435, USA

[3] University of North Texas, Denton, TX, 76203, USA

Table S1: Summary of stove appliance properties of stoves tested in this study. The stove designation refers to the stove technology, with associated specifications. Higher Heating Value Efficiency (%) was collected from the publicly available certification test report. Device 21 was manufactured before the NSPS mandated certification testing and therefore no efficiency is reported. Devices 14A and 32 are pellet stoves and no firebox volume is available to report. The SLM efficiency is calculated using Canadian Standard Association B415.1-10 standard for performance testing of solid biofuel burning heating appliances.

**Device Number**	**Device Type**	**Certification**	**Control**	**Firebox Volume** (ft3)	**Firebox Height** (in)	**Firebox Depth** (in)	**Firebox Width** (in)	**HHV Efficiency** (%)	**SLM Efficiency (%)**
7A	Wood Stove	Step 2	Hybrid	1.90	*	*	*	77.3	74.3
12	Wood Stove	Step 2	Hybrid	2.89	14.75	26	13	76.6	74.9
14A	Pellet Stove	Step 2	N/A	N/A	N/A	N/A	N/A	79.5	85.0
16	Wood Stove	Step 2	NC Tube	2.14	11	16	21	72	65.7
21	Wood Stove	Pre-NSPS	None	2.80	15	25	13	N/A	70.3
26	Wood Stove	Step 2	NC Tube	2.51	12	16.5	22.5	72.8	72.9
32	Pellet Stove	Step 2	N/A	N/A	N/A	N/A	N/A	74.6	80.1

Table S2: Summary of results from test fuel characterization for wood combusted in this study. NESCAUM submitted samples of wood test fuels to Timber Products Inspection, an IAS Accredited Testing Laboratory, for analysis. Three samples (n=3) of maple cordwood, two samples (n=2) of birch cordwood, and a sample each of (n=1) hardwood pellets and (n=1) softwood pellets were analyzed. The average results are detailed in the table. All metals values are reported in mg/kg and were analyzed following the ISO 16967/16968 test method. A dash (-) means this parameter was not measured by the method.

Fuel Properties	**Birch Cordwood**	**Maple Cordwood**	**Hardwood Pellets**	**Softwood Pellets**
Total Moisture (%)	25	16.14	6.11	4.54
Ash (%)	0.21	0.26	0.24	0.5
GCV (BTU/lb)	8383.5	8444.33	8460	8673
NCV (BTU/lb)	7869.5	7927.33	-	-
Volatiles (%)	-	-	84.99	84.7
Carbon (%)	49.6	49.89	49.81	50.99
Hydrogen (%)	5.55	5.58	5.86	5.85
Nitrogen (%)	0.19	0.17	0.23	0.12
Oxygen (%)	44.46	44.09	43.59	42.79
Sulfur (%)	0.01	0.01	0.01	0.01
Arsenic (mg/kg)	<0.01	<0.01	0.01	0.02
Cadmium (mg/kg)	0.23	0.13	0.04	0.02
Calcium (mg/kg)	-	-	980	351
Chromium (mg/kg)	<1	<1	<1	3.59
Iron (mg/kg)	-	-	34.1	151.3
Lead (mg/kg)	3.75	0.92	0.54	0.2
Magnesium (mg/kg)	-	-	216	<100
Manganese (mg/kg)	-	-	37.2	26
Nickel (mg/kg)	<1	<1	<1	2.01
Potassium (mg/kg)	-	-	943	349
Sodium (mg/kg)	-	-	12	75
Zinc (mg/kg)	19.55	3.26	14.21	2.36

Table S3: Cumulative mean VOC emissions factors (EF, mg/kg dry wood) plus the range [min, max] (measured by EPA TO-15A) over the total stove run time. There were 3 VOC samples per cordwood IDC run grouped by load/phase – 1. startup + high fire loads (L1+L2), 2. maintenance load phase (L3), and 3. overnight load burn (L4). There were 4 VOC samples per pellet IDC run grouped by load/phase: 1. L1 (start-up), 2. L2+L3+L4 (total time 70 min), 3. L5+L6 (high setting), 4. L7 (medium setting), to capture the startup phase (L1) plus various IDC settings. The values at the "x" subscript show the percentage of samples below the MDL (and the MDL substituted into the EF calculation). The values flagged at the "y" show the percentage of samples that were outside the high calibration range (with value substituted into the EF calculation). With this information, the reader can interpret the confidence of the result. For example, *uncertified – chloroethane* emissions, x= 30, which indicates 30% of the 30 measurements were <MDL and substituted with the chloroethane MDL value in the EF calculation. Many chlorinated VOCs were either not detected (ND) or more than 60% of the total samples were under the MDL for all stove technologies so not reported, including the following compounds: 1,1-dichloroethene, tetrachloroethylene, trichloroethylene, 1,1,2-trichloroethane, 1,2-dichloroethane, chlorobenzene, and m, p, and o- dichlorobenzene.

Species	**Emission Factors (mg/kg) - Mean [range]$_{x, y*}$**			
	Uncertified (n=10)	Non-Catalytic (n=9)	Catalytic/Hybrid (n=9)	Pellet (n=6)
1,2,4-Trimethylbenzene	5.87 [0.79, 17.32]$_{0,0}$	2.62 [1.18, 6.75]$_{0,0}$	2.26 [1.52, 5.31]$_{11,0}$	1.48 [1.35, 1.68]$_{83,0}$
1,3,5-Trimethylbenzene	2.67 [0.42, 5.31]$_{0,0}$	1.29 [0.63, 3.59]$_{22,0}$	1.10 [0.90, 1.65]$_{33,0}$	100% < MDL
1,3-Butadiene†	290 [206, 402]$_{5,0}$	96.2 [30.6, 158.9]$_{0,0}$	54.9 [41.5, 76.9]$_{0,0}$	6.40 [4.79, 10.77]$_{0,0}$
Acrolein†	637 [422, 812]$_{0,0}$	283 [154, 345]$_{0,0}$	186 [126, 267]$_{0,0}$	42.4 [22.5, 65.2]$_{0,4}$
Acrylonitrile	12.9 [8.6, 17.6]$_{3,3}$	6.5 [3.5, 8.8]$_{0,0}$	4.1 [1.4, 13.7]$_{41,0}$	*100% < MDL*
Alpha-Pinene	0.75 [0.37, 1.81]$_{7,0}$	1.15 [0.56, 2.35]$_{11,0}$	4.50 [0.81, 12.07]$_{7,0}$	*100% < MDL*
Benzene†	1523 [1039, 1964]$_{0,0}$	430 [279, 715]$_{0,0}$	279 [168, 416]$_{0,0}$	30.0 [20.6, 38.6]$_{0,0}$
Butane†	37.3 [6.9, 71.8]$_{0,0}$	50.8 [29.6, 112.1]$_{0,0}$	345 [127, 872]$_{0,0}$	10.7 [0.6, 32.7]$_{0,17}$
Carbon Tetrachloride	0.24 [0.05, 0.83]$_{13,0}$	0.37 [0.08, 0.86]$_{4,0}$	2.18 [0.24, 5.75]$_{0,0}$	1.11 [1.03, 1.20]$_{83,0}$
Chloroethane	0.23 [0.08, 0.42]$_{30,0}$	0.33 [0.11, 0.48]$_{19,0}$	0.45 [0.24, 0.57]$_{26,0}$	0.31 [0.18, 0.68]$_{4,0}$
Chloroform†	0.23 [0.17, 0.32]$_{95,0}$	0.37 [0.27, 0.44]$_{83,0}$	0.56 [0.39, 0.97]$_{11,0}$	19.0 [0.4, 59.4]$_{0,25}$
Chloromethane	12.6 [0.2, 27.7]$_{0,10}$	6.8 [1.6, 15.0]$_{0,0}$	11.3 [2.9, 19.3]$_{0,0}$	1.9 [0.3, 4.1]$_{0,0}$
Cyclohexane	0.71 [0.14, 3.27]$_{10,0}$	0.42 [0.21, 1.09]$_{19,0}$	5.46 [0.30, 27.44]$_{33,0}$	1.10 [0.73, 2.57]$_{50,0}$
Dichloromethane	0.91 [0.30, 2.72]$_{3,0}$	0.78 [0.16, 1.39]$_{4,0}$	1.40 [0.65, 4.62]$_{0,0}$	*100% > HDL*
Ethylbenzene	52.0 [31.3, 73.7]$_{0,13}$	17.2 [4.0, 30.2]$_{0,0}$	13.0 [7.5, 19.5]$_{0,0}$	1.8 [1.0, 2.9]$_{0,0}$
Ethylene Oxide	27.3 [16.7, 43.8]$_{0,7}$	15.2 [6.6, 21.4]$_{0,0}$	11.8 [5.8, 16.1]$_{7,0}$	3.7 [1.5, 5.0]$_{42,0}$
Heptane	2.89 [0.23, 9.93]$_{0,0}$	1.77 [0.51, 5.64]$_{0,0}$	9.94 [0.57, 31.61]$_{0,0}$	1.21 [0.86, 2.06]$_{71,0}$
Hexane	3.95 [0.08, 13.76]$_{0,0}$	4.41 [0.12, 16.95]$_{0,0}$	6.29 [0.17, 32.85]$_{0,0}$	13.3 [2.0, 26.2]$_{0,0}$
m&p-Xylenes	61.7 [46.8, 76.9]$_{0,10}$	22.8 [2.3, 45.6]$_{0,0}$	17.9 [4.7, 29.8]$_{0,0}$	5.7 [1.1, 10.9]$_{0,0}$
n-Butylbenzene	2.73 [0.90, 6.23]$_{0,0}$	1.43 [0.81, 2.32]$_{37,0}$	1.50 [1.16, 2.38]$_{74,0}$	*100% < MDL*
Octane	2.37 [0.27, 7.83]$_{3,0}$	1.56 [0.50, 4.93]$_{0,0}$	3.80 [0.65, 10.11]$_{7,0}$	*100% < MDL*
o-Xylene	26.2 [17.5, 40.4]$_{0,7}$	9.2 [1.2, 18.5]$_{0,0}$	6.8 [2.6, 11.1]$_{0,0}$	2.0 [0.9, 3.5]$_{17,0}$
Pentane	9.1 [1.7, 16.7]$_{0,0}$	8.9 [0.3, 29.1]$_{4,0}$	91.7 [1.9, 362.1]$_{0,0}$	6.6 [0.5, 23.9]$_{0,0}$
Styrene†	189 [149, 259]$_{0,0}$	81.4 [40.7, 140.1]$_{0,0}$	20.1 [0.7, 45.6]$_{0,0}$	1.7 [1.1, 2.6]$_{0,0}$
Toluene†	422 [290, 525]$_{0,0}$	112 [43, 194]$_{0,0}$	88 [44, 151]$_{0,0}$	11.7 [5.2, 17.0]$_{0,0}$
Trichlorofluoromethane	0.40 [0.23, 0.76]$_{3,0}$	0.58 [0.37, 1.18]$_{0,0}$	1.62 [0.56, 6.80]$_{0,0}$	2.90 [0.59, 9.68]$_{0,0}$

* x and y show percentage of the measurements substituted with the corresponding MDL value and high threshold value, respectively.

† For these specific compounds only, the actual number of IDC runs is found by subtracting 3 from n, using the value of n shown next to the cordwood stove technology name. For example, for the toluene EF from the non-catalytic category, there were 6 total IDC runs. For the pellet stove technology, n is equal to 6 IDC runs for all species.

Table S4: Cumulative mean PAH emissions factors (EF, mg/kg dry wood) plus the range [min, max] (measured by EPA TO-13A) over the total stove run time following the IDC test method per cordwood stove technology type. The total runs per stove technology type are shown at the top of the column. Three PAH sample groupings were collected per cordwood IDC run, 1. at conditions of start-up +high fire loads (L1+L2), 2. maintenance load phase (L3) and 3. overnight load phase (L4). All PAHs presented below were detected above the MDL for each sample. To calculate the total samples per PAH, we use as an example benzo (a) pyrene in uncertified stoves. Benzo (a) pyrene was detected in 7 IDC runs times 3 samples per run, for a total of 21 samples. Pellet stove operation did not follow the IDC protocol, and few PAHs were detected. Four total pellet stove samples had detectable levels of naphthalene, 2-methylnapthalene, 1-methylnapthalene, and acenaphthylene, all PAHs detected resulted in EFs <than 1 mg/kg. No other PAHs were detected in pellet samples.

Species	**Emission Factors (mg/kg) - Mean [range]**		
	Uncertified (n=7)	*Non-Catalytic (n=9)*	*Catalytic/Hybrid (n=6)*
Total PAHs	426 [324, 589]	77.1 [36.1, 108.8]	67.8 [54.7, 75.5]
1-Methylnaphthalene	11.9 [9.2, 15.9]	3.60 [1.71, 5.17]	3.38 [2.69, 3.92]
2-Methylnaphthalene	16.2 [12.6, 21.7]	5.14 [2.57, 7.31]	4.59 [3.51, 5.43]
Acenaphthene	2.66 [1.57, 4.26]	0.44 [0.10, 0.77]	0.40 [0.24, 0.53]
Acenaphthylene	35.5 [22.3, 55.6]	6.81 [1.65, 12.26]	6.46 [4.03, 8.70]
Anthracene	12.8 [10.0, 17.8]	1.88 [0.59, 3.89]	1.28 [1.11, 1.73]
Benzo(a)anthracene	7.00 [5.16, 10.10]	1.01 [0.39, 1.72]	0.90 [0.67, 1.36]
Benzo(a)pyrene	7.02 [5.06, 10.58]	0.77 [0.20, 1.30]	0.76 [0.49, 1.24]
Benzo(b)fluoranthene	7.00 [5.00, 10.11]	0.99 [0.35, 1.46]	0.90 [0.62, 1.46]
Benzo(e)pyrene	3.08 [2.14, 4.43]	0.41 [0.13, 0.62]	0.39 [0.26, 0.63]
Benzo(g,h,i)perylene	3.76 [2.78, 5.61]	0.45 [0.12, 0.85]	0.42 [0.25, 0.74]
Benzo(k)fluoranthene	2.42 [1.75, 3.40]	0.31 [0.10, 0.47]	0.31 [0.20, 0.50]
Chrysene	6.21 [4.42, 8.80]	0.99 [0.41, 1.57]	0.85 [0.64, 1.33]
Coronene	1.60 [1.13, 2.48]	0.18 [0.03, 0.38]	0.17 [0.07, 0.30]
Dibenz(a,h)anthracene	0.47 [0.32, 0.72]	0.05 [0.03, 0.11]	0.05 [0.04, 0.06]
Fluoranthene	25.1 [19.2, 34.4]	4.50 [2.14, 6.85]	3.61 [2.86, 5.20]
Fluorene	12.2 [7.3, 18.4]	1.9 [0.4, 3.9]	1.4 [0.9, 2.0]
Indeno(1,2,3-cd)pyrene	3.38 [2.45, 4.91]	0.41 [0.11, 0.65]	0.39 [0.24, 0.66]
Naphthalene	189 [139, 256]	35.5 [20.4, 45.4]	32.5 [25.8, 37.3]
Perylene	1.10 [0.80, 1.67]	0.10 [0.05, 0.17]	0.11 [0.06, 0.20]
Phenanthrene	51.1 [42.0, 65.2]	7.38 [2.79, 12.67]	5.17 [4.42, 7.03]
Pyrene	26.3 [20.3, 37.0]	4.29 [1.83, 6.69]	3.77 [2.89, 5.57]

Table S5: Cumulative mean metals emissions factors (EF, mg/kg db) plus the range [min, max] (measured by ICP/MS) over the total stove run time following the cordwood IDC. Three samples were collected per IDC run at conditions of 1. start-up + high fire loads (L1+L2), maintenance load phase (L3) and overnight load phase (L4). The total number of IDC runs with all samples above the MDL is shown by the number, "n" subscripted near the brackets. For the total number of samples collected per metal, multiply 3 times the subscripted "n", representing the valid IDC runs. Values < MDL were not substituted with the MDL in this table (this resulted in some runs not being included). Pellet stoves did not follow the IDC test protocol but consisted of multiple 4-hour sampling periods at varying operational settings (high, medium, low), which were subsequently pooled and averaged. For pellet stoves, there was a maximum of n=24 samples with measurements above the MDL (see Sections 2.2.2 and 2.3 for more details of the pellet operation).

Species	**Emission Factors (mg/kg) - Mean [range]n***			
	Uncertified	*Non-Cat*	*Cat/Hybrid*	*Pellet*
Sodium	0.60 [0.28, 1.36]$_7$	0.46 [0.16, 0.73]$_3$	0.55 [0.29, 1.16]$_4$	9.65 [4.05, 15.27]$_{24}$
Magnesium	0.18 [0.12, 0.27]$_6$	0.07 [0.06, 0.51]$_9$	0.25 [0.10, 0.56]$_6$	1.23 [0.01, 4.88]$_{13}$
Potassium	33.87 [16.88, 54.39]$_7$	9.91 [6.08, 13.92]$_8$	7.94 [3.82, 12.11]$_6$	208 [54, 397]$_{24}$
Calcium	0.82 [0.56, 1.01]$_6$	3.10 [0.52, 11.98]$_5$	2.42 [0.18, 5.80]$_5$	7.32 [0.20, 20.23]$_{14}$
Manganese	0.10 [0.05, 0.19]$_7$	0.06 [0.02, 0.17]$_8$	0.07 [0.04, 0.12]$_6$	0.32 [0.08, 1.64]$_{24}$
Iron	0.68 [0.11, 2.53]$_5$	0.36 [0.17, 0.65]$_5$	0.50 [0.10, 0.73]$_4$	0.54 [0.01, 2.19]$_{18}$
Zinc	2.43 [0.77, 5.87]$_7$	0.96 [0.12, 2.18]$_9$	0.78 [0.22, 1.43]$_6$	4.39 [1.00, 8.14]$_{24}$
Nickel	0.03 [0.01, 0.05]$_3$	0.010 [0.001, 0.029]$_4$	0.05 [0.03, 0.09]$_4$	0.0197 [0.0005, 0.0833]$_{10}$
Cadmium	0.11 [0.06, 0.17]$_7$	0.07 [0.02, 0.10]$_8$	0.09 [0.02, 0.21]$_6$	0.005 [0.001, 0.015]$_{24}$
Arsenic	0.001 [0.001, 0.002]$_7$	0.0012 [0.0002, 0.0018]$_8$	0.003 [0.001, 0.010]$_6$	0.010 [0.003, 0.017]$_{24}$
Lead	0.88 [0.21, 1.78]$_7$	0.37 [0.10, 0.67]$_8$	0.47 [0.20, 0.82]$_6$	0.23 [0.04, 0.59]$_{24}$

* Number of runs with all valid measurements per run.

Table S6: Cumulative mean and range of emission factors in terms of the mass unit of emission (mg) per unit of stove output energy (MJ). Output energy is calculated using the average SLM efficiencies presented in Table S1. See Table 1 in Section 3.1 for the number of IDC runs per stove technology type.

Species	**Emission Factors (mg/MJ) - Mean [range]**			
	Uncertified	*Non-Cat*	*Cat/Hybrid*	*Pellet*
PM	1064 [774, 1304]	656 [125, 1060]	231 [96, 426]	9.9 [5.4, 14.8]
Black Carbon	36.6 [19.2, 54.8]	13.1 [6.9, 29.8]	15.5 [6.8, 46.1]	0.68 [0.33, 0.86]
Brown Carbon	135 [109, 172]	44.4 [17.3, 70.7]	50.9 [29.2, 124.5]	-
NO	22.7 [19.9, 25.3]	25.3 [22.2, 28.2]	31.0 [18.5, 45.1]	60.2 [43.3, 84.0]
NOx	40.6 [31.8, 66.3]	47.0 [39.3, 65.5]	72.0 [48.5, 95.6]	93.6 [67.9, 125.7]
NO2	7.07 [1.24, 28.11]	9.04 [1.30, 23.11]	24.55 [14.21, 33.00]	1.86 [0.00, 3.35]
CO	6356 [4957, 7880]	3524 [2708, 4046]	1481[1061, 2005	772[636, 971]
CO2	114914 [108856, 120821]	123385 [116863, 133846]	119951 [118378, 121489]	106747 [105558, 108025]
CH4	738 [526, 959]	281 [159, 379]	411 [308, 495]	8.7 [5.0, 17.2]
CH2O	155 [127, 177]	84.5 [52.4, 103.6]	73.5 [47.6, 96.9]	14.9 [12.2, 18.6]
THC	3109 [1574, 4197]	1493.3 [585.3, 1901.4]	1082.9 [845.0, 1458.8]	116.8 [75.7, 143.9]
NMTHC	2378 [953, 3360]	1216 [427, 1531]	675 [403, 1075]	108 [69, 135]

Table S7: Cumulative mean and range of VOCs emission factors in terms of the mass unit of emission (mg) per unit of stove output energy (MJ). Output energy is calculated using the average SLM efficiencies presented in Table S1. See Table S3 for the number of samples, n, per individual VOC.

Species	**Emission Factors (mg/MJ) - Mean [range]**			
	Uncertified	Non-Cat	Cat/Hybrid	Pellet
1,2,4-Trimethylbenzene	0.414 [0.034, 1.254]	0.195 [0.083, 0.523]	0.155 [0.104, 0.364]	0.088 [0.081, 0.105]
1,3,5-Trimethylbenzene	0.180 [0.018, 0.384]	0.093 [0.044, 0.251]	0.075 [0.061, 0.113]	*100% < MDL*
1,3-Butadiene	17.0 [11.1, 22.7]	6.7 [2.1, 11.1]	3.7 [2.8, 5.2]	0.377 [0.292, 0.604]
Acrolein	37.9 [27.9, 58.3]	19.9 [10.7, 24.3]	12.7 [8.6, 18.1]	2.50 [1.37, 3.66]
Acrylonitrile	0.815 [0.537, 1.277]	0.475 [0.246, 0.683]	0.278 [0.099, 0.939]	*100% < MDL*
Alpha-Pinene	0.051 [0.016, 0.131]	0.085 [0.039, 0.182]	0.306 [0.056, 0.820]	*100% < MDL*
Benzene	87.4 [74.7, 113.2]	30.1 [19.6, 49.9]	19.0 [11.5, 28.3]	1.77 [1.25, 2.17]
Butane	2.40 [0.30, 5.24]	3.56 [2.08, 7.89]	23.5 [8.7, 59.3]	0.615 [0.036, 1.836]
Carbon Tetrachloride	0.017 [0.002, 0.060]	0.028 [0.006, 0.067]	0.148 [0.016, 0.391]	0.066 [0.061, 0.073]
Chloroethane	0.015 [0.006, 0.031]	0.024 [0.009, 0.034]	0.031 [0.016, 0.039]	0.018 [0.011, 0.038]
Chloroform	0.014 [0.008, 0.023]	0.026 [0.019, 0.031]	0.038 [0.026, 0.066]	1.155 [0.022, 3.724]
Chloromethane	0.775 [0.014, 1.990]	0.501 [0.113, 1.082]	0.768 [0.200, 1.322]	0.114 [0.020, 0.232]
Cyclohexane	0.051 [0.006, 0.237]	0.031 [0.014, 0.085]	0.371 [0.021, 1.865]	0.066 [0.044, 0.161]
Dichloromethane	0.062 [0.013, 0.197]	0.057 [0.012, 0.108]	0.096 [0.044, 0.317]	*100% > HDL*
Ethylbenzene	3.16 [2.27, 4.13]	1.26 [0.28, 2.11]	0.886 [0.513, 1.327]	0.105 [0.057, 0.183]
Ethylene Oxide	1.67 [1.20, 2.29]	1.11 [0.46, 1.55]	0.808 [0.395, 1.107]	0.222 [0.089, 0.290]
Heptane	0.205 [0.010, 0.719]	0.132 [0.036, 0.437]	0.676 [0.039, 2.149]	0.072 [0.048, 0.119]
Hexane	0.285 [0.004, 0.997]	0.341 [0.008, 1.314]	0.431 [0.012, 2.251]	0.796 [0.119, 1.643]
m&p-Xylenes	3.89 [2.13, 5.00]	1.67 [0.16, 3.19]	1.22 [0.32, 2.03]	0.338 [0.064, 0.682]
n-Butylbenzene	0.182 [0.066, 0.451]	0.105 [0.057, 0.179]	0.103 [0.079, 0.163]	*100% < MDL*
Octane	0.167 [0.012, 0.567]	0.117 [0.035, 0.382]	0.259 [0.045, 0.687]	*100% < MDL*
o-Xylene	1.66 [0.94, 2.93]	0.669 [0.086, 1.293]	0.462 [0.178, 0.763]	0.121 [0.055, 0.220]
Pentane	0.623 [0.071, 1.216]	0.638 [0.023, 2.046]	6.241 [0.132, 24.617]	0.401 [0.028, 1.496]
Styrene	11.1 [7.8, 15.1]	5.71 [2.84, 9.79]	1.37 [0.04, 3.12]	0.101 [0.064, 0.147]
Toluene	24.6 [17.5, 31.8]	7.88 [3.03, 13.57]	5.96 [2.99, 10.25]	0.693 [0.319, 1.065]
Trichlorofluoromethane	0.025 [0.010, 0.035]	0.042 [0.028, 0.083]	0.110 [0.038, 0.462]	0.175 [0.035, 0.607]

Table S8: Cumulative mean and range of PAHs emission factors in terms of the mass unit of emission (mg) per unit of stove output energy (MJ). Output energy is calculated using the average SLM efficiencies presented in Table S1. See Table S4 for the number of samples n, for each PAH.

Species	**Emission Factors (mg/MJ) - Mean [range]**		
	Uncertified	*Non-Cat*	*Cat/Hybrid*
Total PAHs	31.1 [23.6, 43.0]	5.5 [2.8, 7.6]	4.6 [3.7, 5.2]
1-Methylnaphthalene	0.9 [0.7, 1.2]	0.26 [0.13, 0.36]	0.23 [0.18, 0.27]
2-Methylnaphthalene	1.2 [0.9, 1.6]	0.37 [0.20, 0.51]	0.31 [0.24, 0.37]
Acenaphthene	0.19 [0.11, 0.31]	0.03 [0.01, 0.05]	0.03 [0.02, 0.04]
Acenaphthylene	2.6 [1.6, 4.1]	0.49 [0.13, 0.86]	0.44 [0.27, 0.59]
Anthracene	0.9 [0.7, 1.3]	0.13 [0.05, 0.27]	0.09 [0.08, 0.12]
Benzo(a)anthracene	0.51 [0.38, 0.74]	0.07 [0.03, 0.12]	0.06 [0.05, 0.09]
Benzo(a)pyrene	0.51 [0.37, 0.77]	0.06 [0.02, 0.09]	0.05 [0.03, 0.08]
Benzo(b)fluoranthene	0.51 [0.36, 0.74]	0.07 [0.03, 0.10]	0.06 [0.04, 0.10]
Benzo(e)pyrene	0.22 [0.16, 0.32]	0.03 [0.01, 0.04]	0.03 [0.02, 0.04]
Benzo(g,h,i)perylene	0.27 [0.20, 0.41]	0.03 [0.01, 0.06]	0.03 [0.02, 0.05]
Benzo(k)fluoranthene	0.18 [0.13, 0.25]	0.02 [0.01, 0.03]	0.02 [0.01, 0.03]
Chrysene	0.45 [0.32, 0.64]	0.07 [0.03, 0.11]	0.06 [0.04, 0.09]
Coronene	0.12 [0.08, 0.18]	0.01 [0.00, 0.03]	0.01 [0.01, 0.02]
Dibenz(a,h)anthracene	0.03 [0.02, 0.05]	0.00 [0.00, 0.01]	0.00 [0.00, 0.00]
Fluoranthene	1.8 [1.4, 2.5]	0.32 [0.17, 0.48]	0.25 [0.20, 0.35]
Fluorene	0.9 [0.5, 1.3]	0.14 [0.03, 0.27]	0.10 [0.06, 0.13]
Indeno(1,2,3-cd)pyrene	0.25 [0.18, 0.36]	0.03 [0.01, 0.05]	0.03 [0.02, 0.04]
Naphthalene	13.8 [10.2, 18.7]	2.6 [1.6, 3.2]	2.2 [1.8, 2.6]
Perylene	0.08 [0.06, 0.12]	0.01 [0.00, 0.01]	0.01 [0.00, 0.01]
Phenanthrene	3.7 [3.1, 4.8]	0.53 [0.22, 0.88]	0.35 [0.30, 0.48]
Pyrene	1.9 [1.5, 2.7]	0.31 [0.14, 0.47]	0.26 [0.20, 0.38]

Table S9: Cumulative mean and range of metal emission factors in terms of the mass unit of emission (mg) per unit of stove output energy (MJ). Output energy is calculated using the average SLM efficiencies presented in Table S1. See Table S4 for the number of samples, n, per individual species.

Species	**Emission Factors (mg/MJ) - Mean [range]**			
	Uncertified	*Non-Cat*	*Cat/Hybrid*	*Pellet*
Sodium	0.044 [0.021, 0.099]	0.034 [0.011, 0.055]	0.037 [0.020, 0.080]	0.60 [0.26, 0.95]
Magnesium	0.013 [0.008, 0.020]	0.012 [0.004, 0.036]	0.017 [0.007, 0.038]	0.08 [0.00, 0.30]
Potassium	2.5 [1.2, 4.0]	0.72 [0.43, 1.05]	0.54 [0.26, 0.82]	13.1 [3.3, 25.2]
Calcium	0.060 [0.041, 0.074]	0.22 [0.04, 0.84]	0.165 [0.012, 0.397]	-
Manganese	0.007 [0.004, 0.014]	0.005 [0.001, 0.012]	0.005 [0.003, 0.008]	0.020 [0.005, 0.104]
Iron	0.050 [0.008, 0.184]	0.026 [0.012, 0.046]	0.034 [0.007, 0.050]	0.034 [0.000, 0.139]
Zinc	0.178 [0.056, 0.428]	0.069 [0.009, 0.154]	0.053 [0.015, 0.098]	0.277 [0.062, 0.516]
Nickel	0.002 [0.001, 0.004]	0.001 [0.000, 0.002]	0.003 [0.002, 0.006]	0.0012 [0.0000, 0.0053]
Cadmium	0.008 [0.005, 0.012]	0.005 [0.001, 0.007]	0.006 [0.001, 0.014]	0.0003 [0.0001, 0.0010]
Arsenic	0.0001 [0.0000, 0.0001]	0.00008 [0.00002, 0.00012]	0.0002 [0.0001, 0.0007]	0.0006 [0.0002, 0.0011]
Lead	0.064 [0.015, 0.131]	0.026 [0.007, 0.047]	0.032 [0.013, 0.056]	0.015 [0.003, 0.038]

Table S10: Results of one-way Kruskal Wallis ANOVA with Dunn's post hoc tests comparing stove technologies for key criteria pollutants, greenhouse gases, VOCs, and PAHs. We adjusted the p-values for multiple comparisons using Benjamini-Hochberg procedure to maintain a balance between Type I error control and statistical power. The matrix shows the mean rank difference of EFs between stove technology groups, and their significance, if any. Pollutant names were shown here only when the Kruskal-Wallis resulted in a significant difference in at least one comparison. Hence, other pollutants not shown were not significantly different between technologies at $p < 0.05$. For example, toxic elemental metals (As, Pb, Cd, Mn) were not significantly different between cordwood stove technologies and are thus not shown. Pellet EFs were not included in the PAH or elemental metals Kruskal-Wallis test due to a different testing protocol than the IDC for these compound categories (see Section 2.2.1 and 2.3 for more details). Significance levels of the Dunn's test: ***, **, and * denote p<0.01 (red color highlight), p< 0.05 (orange color), and p<0.10 (yellow color), respectively.

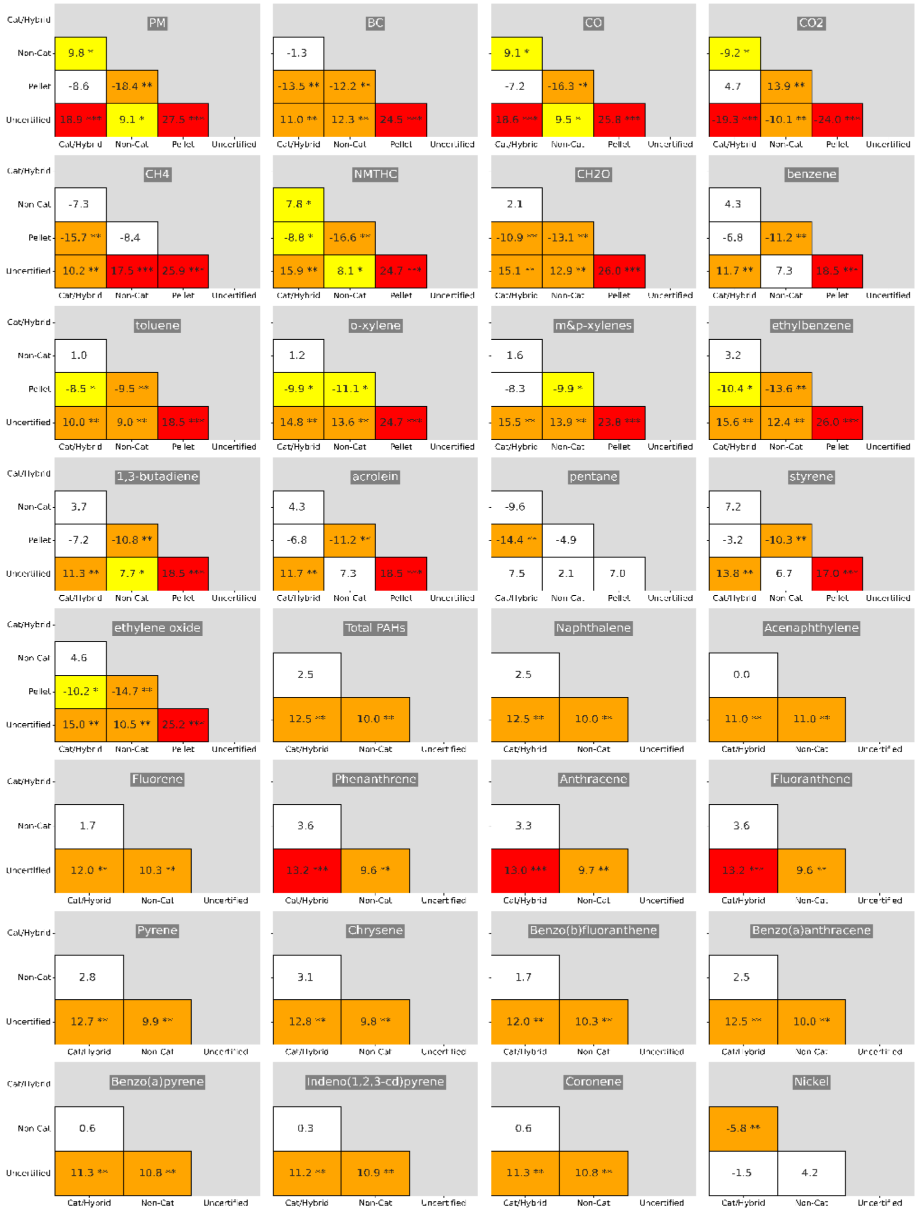

Table S11: Comparison of this study's results to comparable studies in the scientific literature for PM, CO, CO_2, CH_4, NOx, eBC (BC), OC, and Total PAH. Units are g/kg db unless otherwise noted. A dash means the data was not reported or measured in the referenced study.

g/kg db unless noted	***This Study***				**Alves et al. 2011**	**Bhattu et al. 2019**		**Bruns et al. 2015**	**Fine et al. 2004**		**McDonald et al. 2000**	**ECCC 2023**				**Tissari et al. 2007**
Device Type	Un-certified Wood Stove	Catalytic Wood Stove	Non-Catalytic Wood Stove	Pellet Stove	Non-Catalytic Wood Stove	Single-Stage Wood Stove	Pellet Stove	Non-Catalytic Wood Stove	Catalytic Wood Stove (not engaged)	Catalytic Wood Stove	Non-Catalytic Wood Stove	Pre-NSPS Wood Stove	Catalytic Wood Stove	Non Catalytic Wood-stove	Pellet Stove	Stove (soapstone)
Fuel Type	*Hard-woods (Birch, Red Maple)*	*Hard-woods (Birch, Red Maple)*	*Hard-woods (Birch, Red Maple)*	*Hard-wood & Softwood Pellets*	Portugese Oak		Mixed Pellets	Beech	White Oak	White Oak	Mixed Hard-wood	Mixed Hard-wood and Soft-wood	Mixed Hard-wood and Soft-wood	Mixed Hard-wood and Soft-wood	Mixed Pellets	Spruce
Total PM EF	14.62	3.38	8.98	0.86	9.8	POA*	POA*	0.3	3.4	2.2	5.1 g/kg	15.6	2.29	6.99	1.91	0.9 g/kg (as PM1)
CO	87.36	22.67	48.62	13.99	-	49.1	28.8 g/kg	46.7	-	-	123 g/kg	149	26	107	21.5	35 g/kg
CO2	1577	1774	1711	1807	-	1553	1654 g/kg	1738	-	-	-	1580	1570	1902	1922	-
CH4	10.15	6.02	3.9	0.2	-	2.91	0.36 g/kg	3.50	-	-	-	9.83	10.5	5.14	2.19	0.51 g/kg
NOx	0.56	1.05	0.64	1.58	-	-	-	-	-	-	-	0.587	1.58	0.828	2.12	0.48 g/kg
eBC (or EC)	0.5	0.23	0.18	0.012	0.38	0.219	0.05	97.4 mg/kg	0.11	0.23	356.3 mg/kg	0.175	0.129	0.105	0.028	0.61 g/kg
Organic C	-	-	-	-	4.84	-	-	-	1.9	1.2	2821 mg/kg	10.3	1.52	4.31	0.992	-
Total PAH (mg/kg)	426	67.8	77.1	-	-	-	-	10.3	-	-	74.7	118	52	77	10	21 mg/kg

*POA or primary organic aerosol, not PM, was reported

(-) dash means not reported

Table S12: Comparison of this study's results to comparable studies in the scientific literature for PAHs. Units are in mg/kg dry wood burned unless otherwise noted. A dash means the data was not reported or measured in the referenced study.

**Literature, in mg/kg db unless otherwise noted**	**This Study**			**Bhattu et al. 2019**		**Bruns et al. 2015 #**	**Czech et al. 2018 +**	**Fine et al. 2014 ^**		**Hays et al. 2003***	**McDonald et al. 2000**	**Tissari et al. 2007**
**Device Type**	Uncertified Wood Stove	Catalytic Wood Stove	Non-Catalytic Wood Stove	Single-Stage Wood Stove	Pellet Stove	Non-Catalytic Wood Stove	Modern Masonry Heater	Catalytic Stove (not engaged)	Catalytic Wood Stove	Non-Catalytic Wood Stove	Non-Catalytic Wood Stove	Stove
**Fuel Type**	Hardwoods (Birch, Red Maple)	Hardwoods (Birch, Red Maple)	Hardwoods (Birch, Red Maple)	*	Mixed Pellet s	Beech	Birch	White Oak	White Oak	Oak	Mixed Hardwood	Spruce
**Acenaphthene**	2.66	0.4	0.44	-	-	-	-	-	-	-	0.52	-
**Acenaphthylene**	35.5	6.46	6.81	20.61	0.77	-	-	-	-	-	5.19	13.9
**Anthracene**	12.8	1.28	1.88	-	-	-	0.0232	0.032	0.045	-	1.43	-
**Benzo(a)anthracene**	7	0.9	1.01	-	-	-	0.0100	0.147	0.272	0.143	0.56	-
**Benzo(a)pyrene**	7.02	0.76	0.77	-	-	0.094	0.0100	0.112	0.217	0.5673	0.2	-
**Benzo(b)fluoranthene**	7	0.9	0.99	-	-	-	-	0.087	0.211	-	-	0.26
**Benzo(e)pyrene**	3.08	0.39	0.41	-	-	-	0.0087	0.065	0.132	0.36	0.14	-
**Benzo(g,h,i)perylene**	3.76	0.42	0.45	-	-	-	0.0033	0.058	0.119	0.071	0.09	-
**Benzo(k)fluoranthene**	2.42	0.31	0.31	-	-	-	0.0000	0.098	0.227	0.2614	-	0.26
**Chrysene**	6.21	0.85	0.99	-	-	0.121	0.0207	0.179	0.322	-	0.35	-
**Coronene**	1.6	0.17	0.18	-	-	-	<MDL	0.153	0.24	0.024	0.03	-
**Dibenz(a,h)anthracene**	0.47	0.05	0.05	-	-	-	<MDL	0.009	0.017	0.0067	-	-
**Fluoranthene**	25.1	3.61	4.5	-	-	0.05	0.1157	0.562	0.783	0.1971	1.75	-
**Fluorene**	12.2	1.4	1.9	-	-	-	-	-	-	-	1.66	-
**Indeno(1,2,3-cd)pyrene**	3.38	0.39	0.41	-	-	0.067	0.0033	0.077	0.164	0.1379	0.08	0.15
**Naphthalene**	189	32.5	35.5	39.12	0.48	-	0.0000	-	-	-	28.06	-
**Perylene**	1.1	0.11	0.1	-	-	-	0.0004	0.017	0.03	0.089	-	-
**Phenanthrene**	51.1	5.17	7.38	1.71	0.025	-	-	0.194	0.272	-	7.35	-
**Pyrene**	26.3	3.77	4.29	-	-	0.053	0.0958	0.552	0.812	0.1669	1.49	0.75

#Bruns et al. used a denuder before the filter

+Czech et al. 2018 needed conversion factor from birch heating value, soapstone stove

^Fine et al. reports in mg/g of OC (organic carbon) so not a direct comparison to PAH per kg of wood fuel
*Hays et al. 2003 reports only particle phase PAHs captured on aluminum filter substrate
(-) dash means not reported

Table S13: Comparison of this study's results to comparable studies in the scientific literature for VOCs. Units are in mg/kg dry wood burned unless otherwise noted. A dash means the data was not reported or measured in the referenced study. *Italics* means the result was detected but < MDL.

**Literature, mg/kg db unless otherwise noted**	**VOCs, this study**					**Bhattu et al. 2019**		**Cerqueria et al. 2017**	**McDonald et al. 2000**	**Tissari et al. 2007**
Device Type	Uncertified Wood Stove	Catalytic Wood Stove	Non-Catalytic Wood Stove	Pellet Stove	Pellet Stove	Single-Stage Wood Stove	Pellet Stove	Non-Catalytic Wood Stove	Non-Catalytic Wood Stove	Stove (soapstone)
Fuel Type	Hardwoods (Birch, Red Maple)	Hardwoods (Birch, Red Maple)	Hardwoods (Birch, Red Maple)	All Pellets (pooled)	All Pellets (pooled)	Beech	Mixed Pellets	Pyrenean Oak	Mixed Hardwood	Spruce
Acrylonitrile	12.9	4.1	6.5	0.4		8.3	0.233	-	-	-
Benzene	1523	279	430	30		202	7.87	-	1189.68	390
Formaldehyde	2140	1080	1160	*250*		590	43.15	1775	245.58	350
Octane	2.37	3.8	1.56	1.6		-	-	-	14.94	120
O-xylene	26.2	6.8	9.2	2		-	-	-	27.28	220
Pentane	9.1	91.7	8.9	6.6		-	-	-	13.12	180
Styrene	189	20.1	81.4	1.7		23.2	0.96	-	-	-
Toluene	422	88	112	11.7		61.2	2.39	-	320	-
1, 3- Butadiene	290	54.9	96.2	6.4		96.1	1.8	-	196.58	110

(-) dash means not reported

Table S14: Comparison of this study's results to comparable studies in the scientific literature for metals. Units are in mg/kg dry wood burned unless otherwise noted. A dash means the data was not reported or measured in the referenced study.

Literature, mg/kg db	***This Study***				**Alves et al. 2011**	**Czech et al. 2018**	**Fine et al. 2004**		**McDonald et al. 2000**	**Tissari et al. 2007**
Device Type	Uncertified Wood Stove	Catalytic Wood Stove	Non-Catalytic Wood Stove	Pellet Stove	Non-Catalytic Wood Stove	Modern Masonry Heater	Non-Catalytic Wood Stove	Catalytic Wood Stove	Non-Catalytic Wood Stove	Stove (soapstone)
Fuel Type	Hardwoods (Birch, Red Maple)	Hardwoods (Birch, Red Maple)	Hardwoods (Birch, Red Maple)	Hardwood & Softwood Pellets	Portugese Oak	Birch	White Oak	White Oak	Mixed Hardwood	Spruce
Sodium	0.6	0.55	0.46	9.65	-	0.954	-	-	1.95	2.51
Magnesium	0.18	0.25	0.17	1.23	33	-	-	-	-	0.11
Potassium	33.87	7.94	9.91	208	12412	0.037	1.514	2.74	15.36	17.7
Calcium	0.82	2.42	3.10	7.32	-	-	-	-	-	0.84
Manganese	0.1	0.07	0.06	0.32	11.6	0.136	<MDL	<MDL	-	-
Iron	0.68	0.5	0.36	0.54	-	0.693	-	-	-	-
Zinc	2.43	0.78	0.96	4.39	-	12.390	0.0055	0.014	-	2.01
Nickel	0.033	0.051	0.01	0.0197	-	-	<MDL	<MDL	-	-
Cadmium	0.11	0.09	0.07	0.005	-	0.045	<MDL	<MDL	-	-
Arsenic	0.001	0.003	0.0012	0.01	-	-	<MDL	<MDL	-	-
Lead	0.88	0.47	0.37	0.23	12.2	0.161	0.0442	0.114	-	1.2 mg/kg

(-) Not Reported

<MDL- under the minimum detection limit

Table S15a: Cordwood stove IDC load/phase summary. IDC protocols assess appliance performance under typical homeowner operating conditions. The method requires three replicates to assess performance repeatability. The IDC Cordwood Stove protocol assesses common homeowner operations previously identified during data logger analysis of use patterns in stoves in multiple homes over multiple years (Ahmadi et al. 2020 [51]). The IDC method assesses how stoves perform under variable conditions including different fuel loading volumes, coal bed conditions, and piece sizes (which impacts air to fuel ratios).

Phase/Load	Purpose	Fuel loading volume	Piece sizes	Air Setting	Coal bed conditions at start of phase	End of phase
L1	Reflects typical homeowner starting a fire in the appliance	4 lb/ft^3	Kindling – pieces ~1/10 lb<br>Starter fuel – pieces generally 1-3 lb	Fully open	none	When 75% of fuel load consumed
L2	Reloading the stove to achieve high heat output assumes homeowner actively managing the stove	7 lb/ft^3	Small pieces (generally 3-5 lb per piece)	Fully open for first 50% of fuel load then low	~1 lb/ft^3	When 90% of L2 fuel load consumed
L3	Reloading the stove to achieve medium heat output assumes homeowner semi-actively managing the stove	5 lb/ft^3	2-3 large pieces (generally >5 lb per piece)	Lowest air setting	~1.7 lb/ft^3	When 90% of L3 fuel load consumed
L4	Reloading the stove to achieve low heat output assumes homeowner not-actively managing the stove (overnight burn or away from home)	12 lb/ft^3	Mix of small and large pieces	Lowest air setting	~2.2 lb/ft^3	When 90% of L4 fuel load consumed

Table S15b: Pellet stove IDC load/phase summary. The Pellet Stove IDC protocol assesses appliance performance under a variety of heat settings and transitions. Analysis of use patterns indicates that pellet stove use is highly dependent on home heating needs. Current test methods do not assess key transition periods which are common and typically higher emitting than steady state performance. The pellet IDC protocol requires the stove to perform in a variety heat settings and heat transitions. The protocol also assesses cold start performance and warm start performance (which reflects typical idling patterns).

Phase/Load	Purpose	Air setting	End of phase
L1	Start-up to high heat	Highest	60 minutes
L2	Transition - high heat to low heat	Lowest	30 minutes
L3	Assess idle emissions short term	Off	10 minutes
L4	Startup to low heat	Low	30 minutes
L5	Transition low to high heat	High	30 minutes
L6	Assess idle emissions – long term	Off	20 minutes
L7	Start-up to medium heat	40-60% of high/low	90 minutes
L8	Medium to low transition	Low	30 minutes

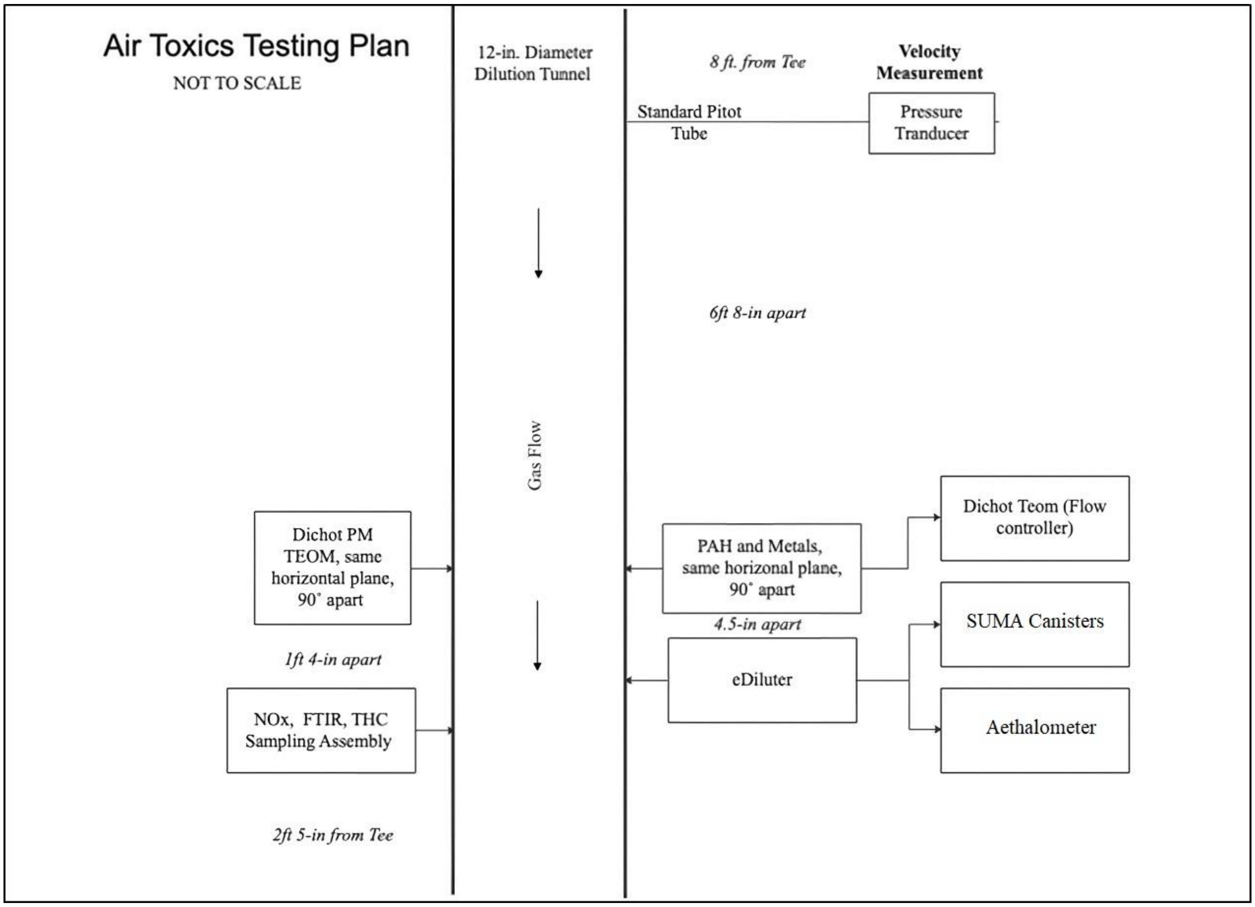

Figure S1: Location of measurement systems in dilution tunnel.

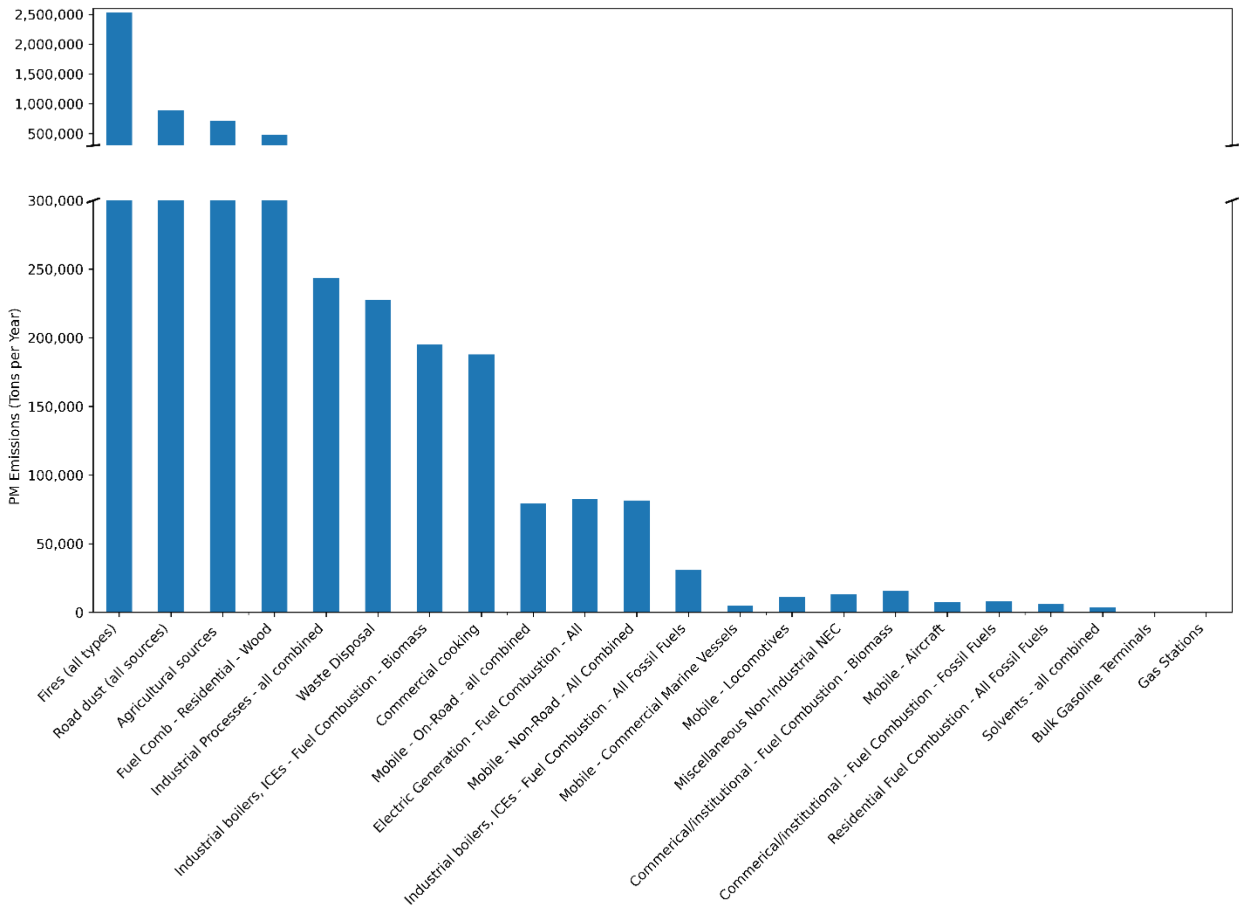

Figure S2: Comparison of EPA NEI 2020 residential wood combustion as a source of $PM_{2.5}$ to other non-point, mobile, and stationary sources (Source: EPA 2020 NEI database).

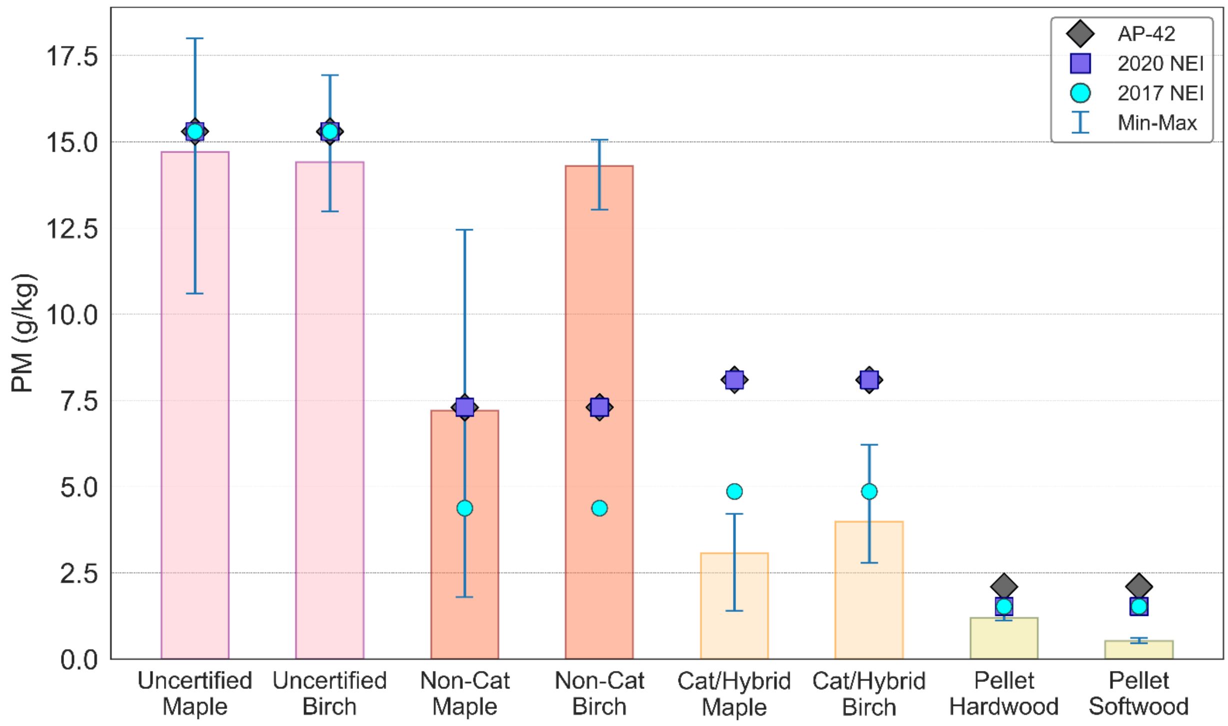

Figure S3A: Plot showing the impact of birch vs. maple wood fuel species on PM emissions for all cordwood stove technologies, and hardwood vs. softwood for pellet stoves. There was no influence of wood fuel type in uncertified and catalytic/hybrid PM EFs. Burning birch cordwood emitted almost 2 times the PM as maple in non-catalytic stoves, exceeding the AP-42, the NEI 2017 and NEI 2020 regulatory reference EFs. Hardwood pellets emitted more than 2 times the PM as softwood pellets.

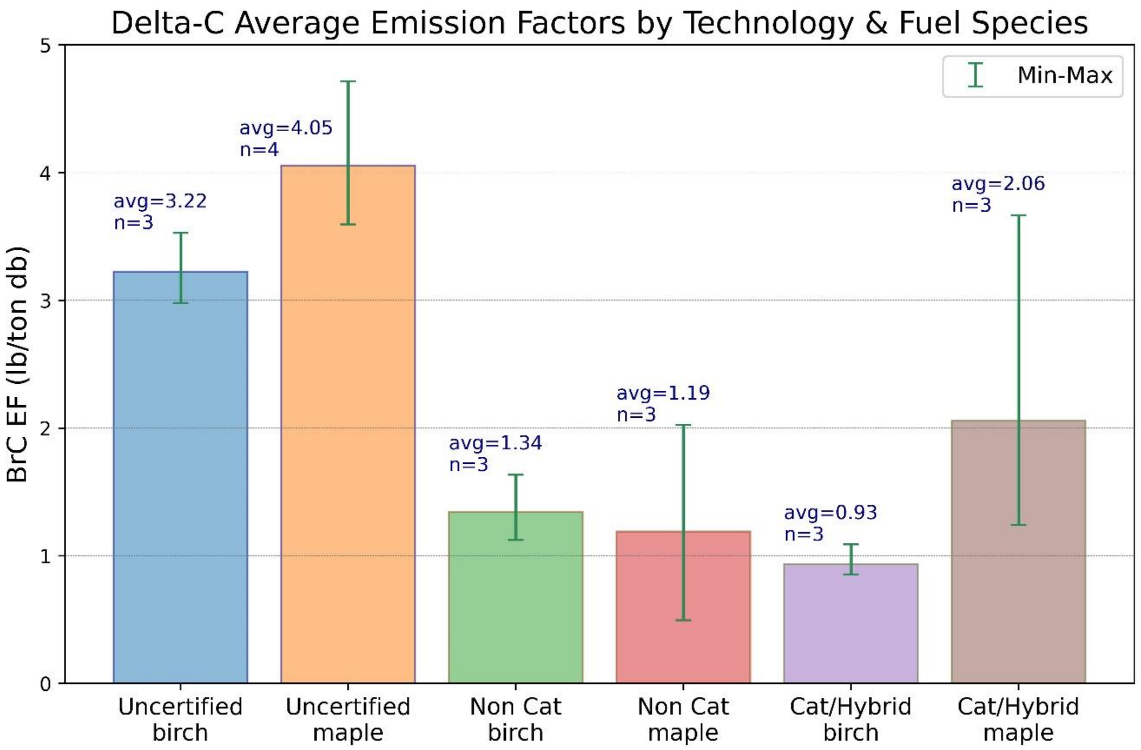

Figure S3B: Plot showing the impact of birch vs. maple wood fuel species on brown carbon or BrC emissions for all cordwood stove technologies. Brown carbon, or BrC, results were estimated as "Delta-C", the difference between the Aethalometer concentrations at 370 nm and 880 nm wavelengths. "Delta-C" is considered a semi-quantitative measure of brown organic carbon aerosol.

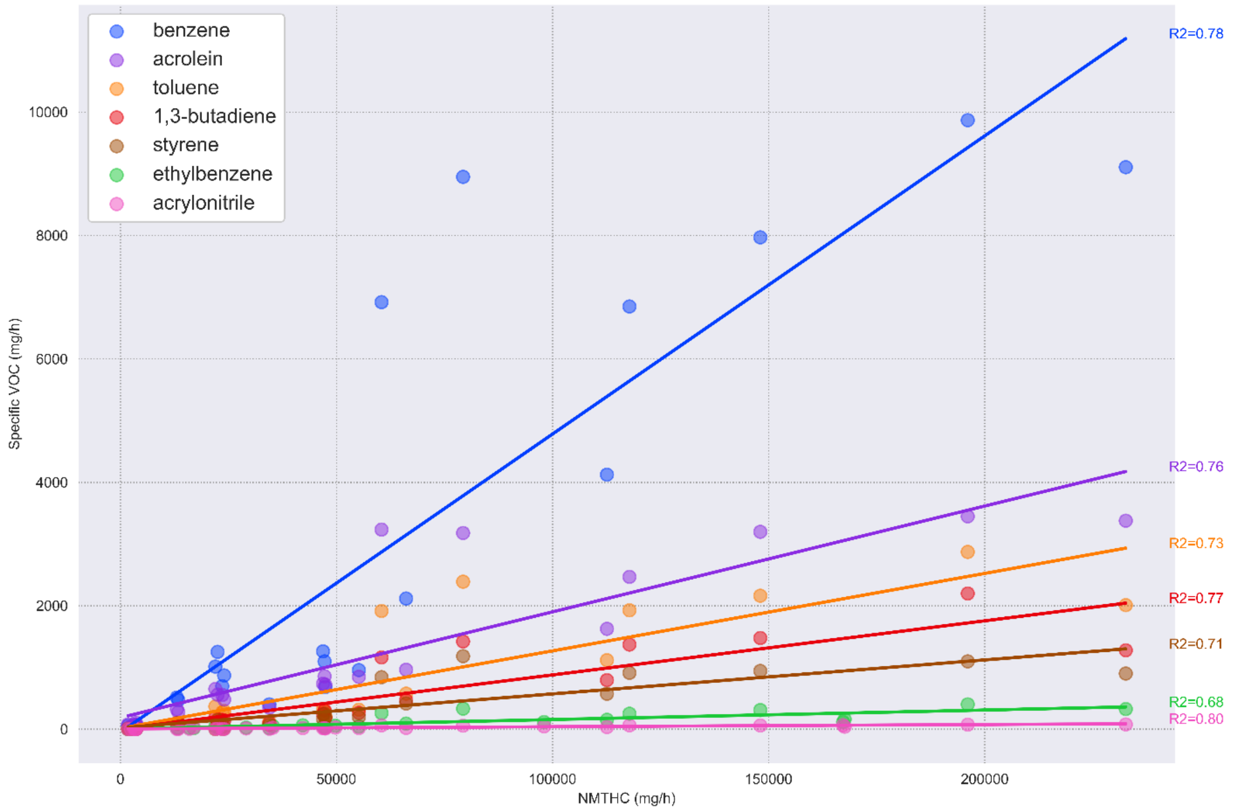

Figure S4: NMTHC (or "total VOC") vs. individual specific VOC species plot, in mg/h. We compared cordwood stove total run average NMTHC emission rates BC (mg/h) (NMTHC determined by "EPA Method 25A THC" minus "EPA Method 320 CH_4") on the x axis, versus total run average individual or "specific VOC" emission rates (mg/h) measured by EPA Method TO-15A on the y-axis. Results show good agreement between the cordwood NMTHC and individual VOC measurements with r^2 ranging from 0.68 to 0.80.

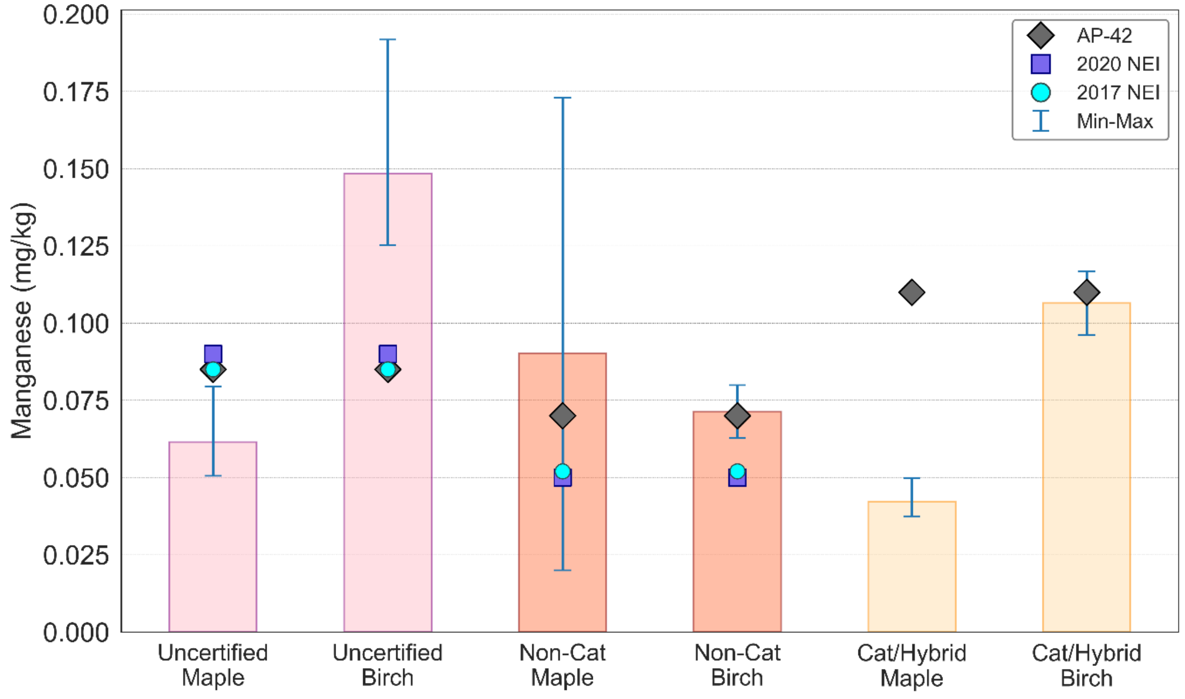

Figure S5: Plot showing the impact of birch vs. maple species on Mn emissions from different cordwood stove technologies. The impact of wood fuel type (birch) was seen in uncertified and catalytic/hybrid Mn EFs, but not in non-catalytic stoves.

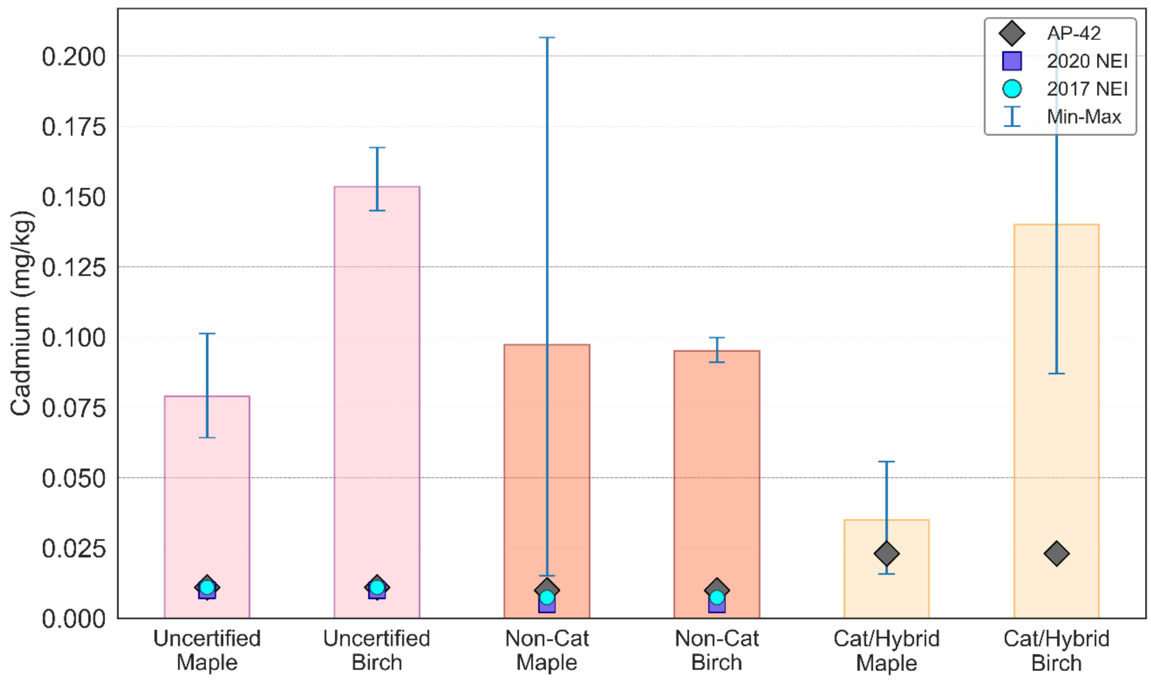

Figure S6: Plot showing the impact of birch vs. maple species on Cd emissions from different cordwood stove technologies. The impact of wood fuel type (birch) was seen in uncertified and catalytic/hybrid Cd EFs, but not in non-catalytic stoves.

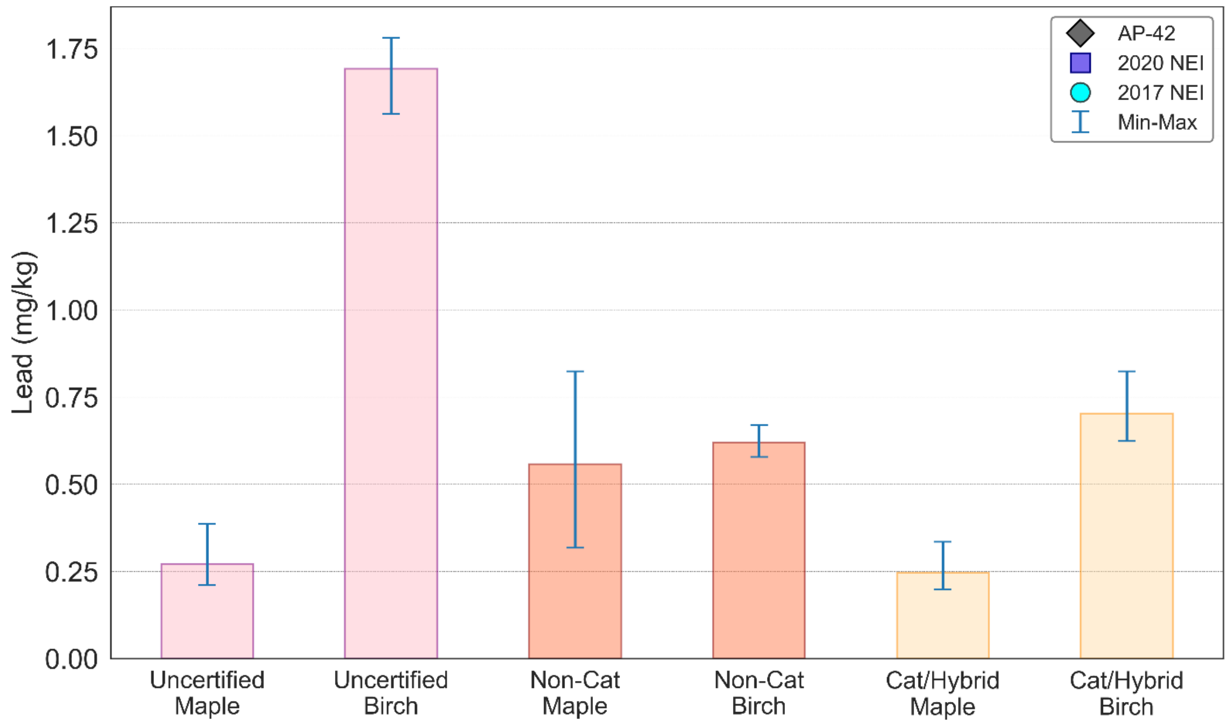

Figure S7: Plot showing the impact of birch vs. maple species on Pb emissions from different cordwood stove technologies. The impact of wood fuel type (birch) was seen in uncertified and catalytic/hybrid Pb EFs but not in non-catalytic stoves.

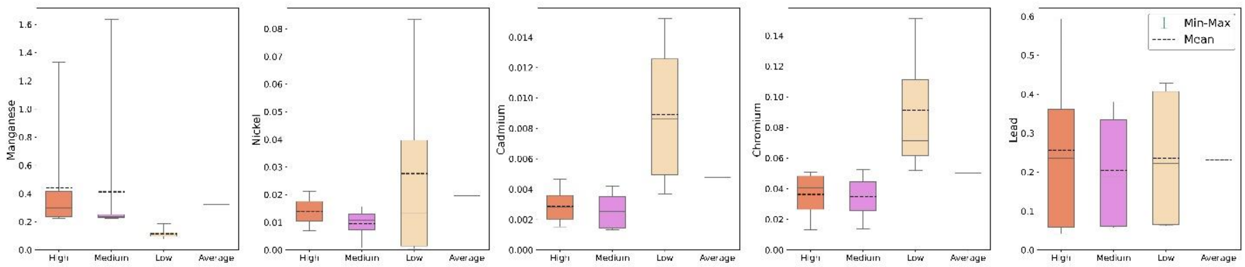

Figure S8: Plot showing Mn, Ni, Cd, Cr and Pb EFs from pellet stoves, in mg/kg wood burned, dry basis, four (4-hour) runs at low, medium, high operational settings. Hardwood and softwood pellet data are pooled, for n=24 samples, except for Ni (n=10) and Cr (n=17)

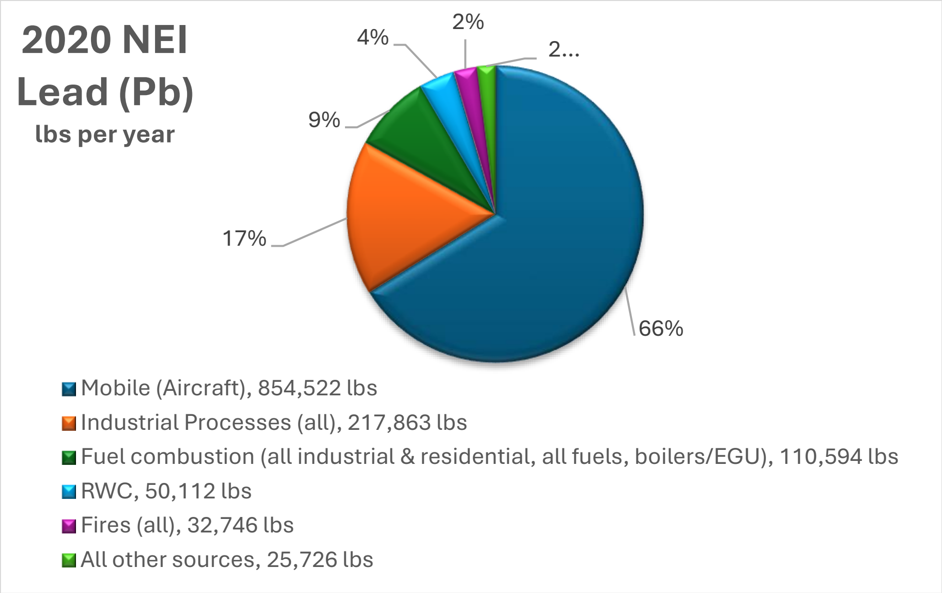

Figure S9: Results of Pb emissions by sector using the 2020 EPA NEI after applying this study's RWH emissions factors. RWH as a category is currently not assessed for Pb in the 2020 NEI, although Pb from other fire categories (i.e., wildfires, prescribed burns) is reported. By applying the Pb EFs from this study, we found that Residential Wood Combustion (RWC) is the 4th largest source category for Pb in the 2020 NEI. To assess the full universe of RWH appliances in the NEI RWC category, in addition to using the Pb EFs per stove category, we applied the uncertified stove Pb EF to uncertified RWH appliances in the Residential Wood Combustion source category (i.e, hydronic heaters, furnaces, fireplaces, and outdoor wood boilers), and the non-catalytic stove Pb EF to certified RWH appliances (see Table S5). This resulted in estimated total Pb emissions from RWC of 50,112 pounds per year, compared to all fires (wildfires, and prescribed) at 32,746 pounds per year.

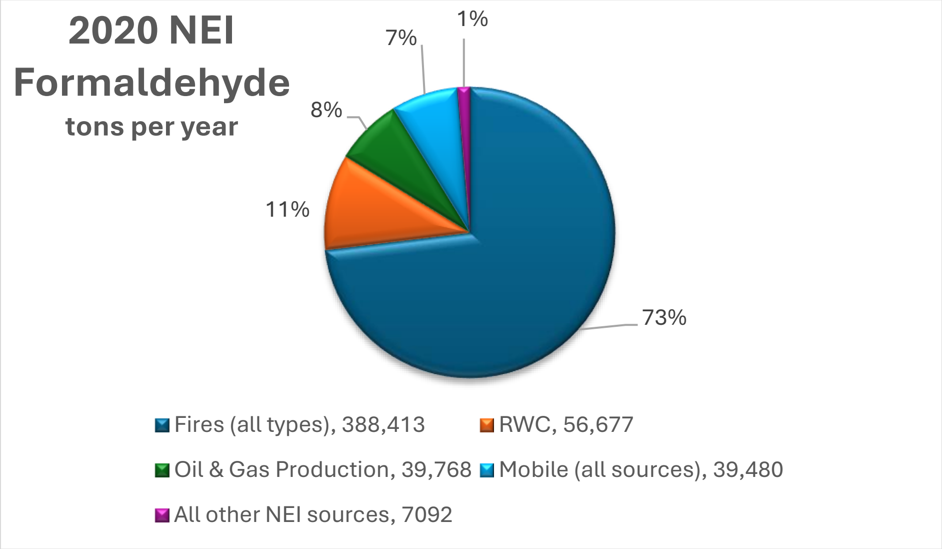

Figure S10: Results of formaldehyde (a 2019 EPA AirToxScreen National Cancer Driver) emissions by sector using the 2020 EPA NEI, in tons per year, after applying this study's emissions factors. After fires (all types, which includes wildfires, prescribed fires, and agricultural field burns), RWC is the 2nd largest source category for formaldehyde in the 2020 NEI.

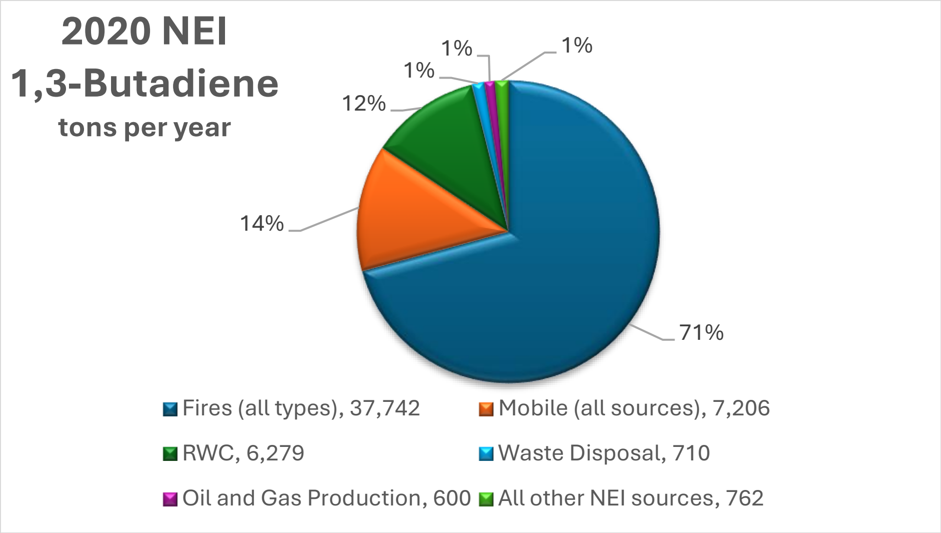

Figure S11: Results of 1,3-butadiene (a 2019 EPA AirToxScreen National Cancer Risk Contributor) emissions by sector using the 2020 EPA NEI, in tons per year, after applying this study's emissions factors. After fires (all types, which includes wildfires, prescribed fires, and agricultural field burns) and mobile sources (all categories, combined), RWC is the 3rd largest source category for 1,3-butadiene in the 2020 NEI.

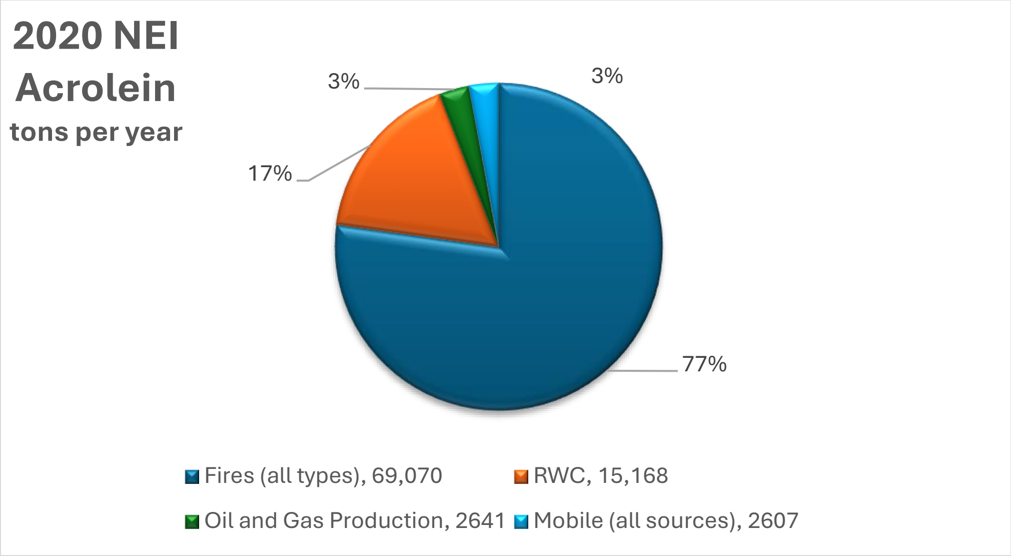

Figure S12: Results of acrolein (an EPA HAP) emissions by sector using the 2020 EPA NEI, in tons per year, after applying this study's emissions factors. After fires (all types, which includes wildfires, prescribed fires, and agricultural field burns) and mobile sources (all categories, combined), RWC is the 3rd largest source category for acrolein in the 2020 NEI.

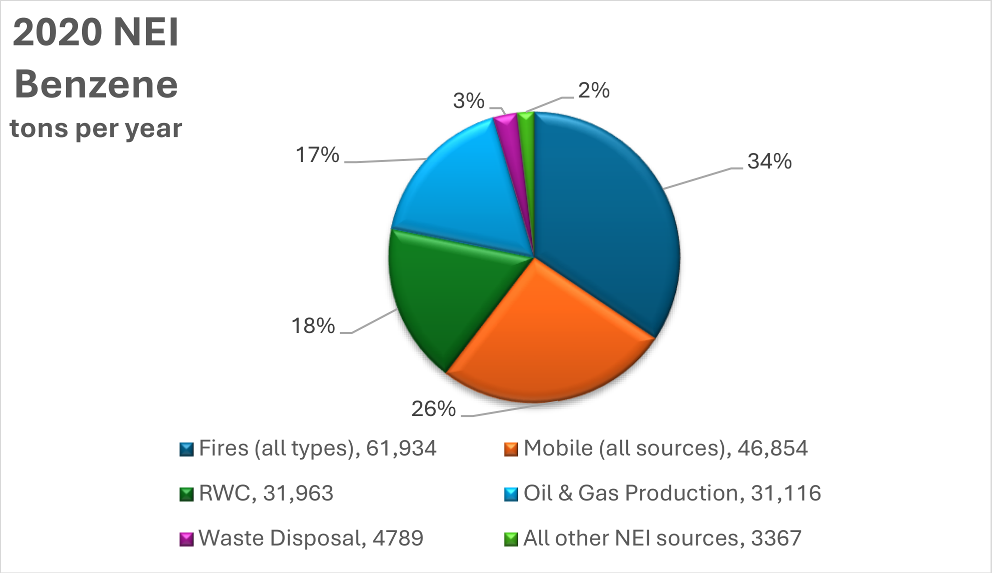

Figure S13: Results of benzene (a 2019 EPA AirToxScreen National Cancer Contributor) emissions by sector using the 2020 EPA NEI, in tons per year, after applying this study's emissions factors. After fires (all types, which includes wildfires, prescribed fires, and agricultural field burns) and mobile sources (all categories, combined), RWC is the 3rd largest source category for benzene in the 2020 NEI.

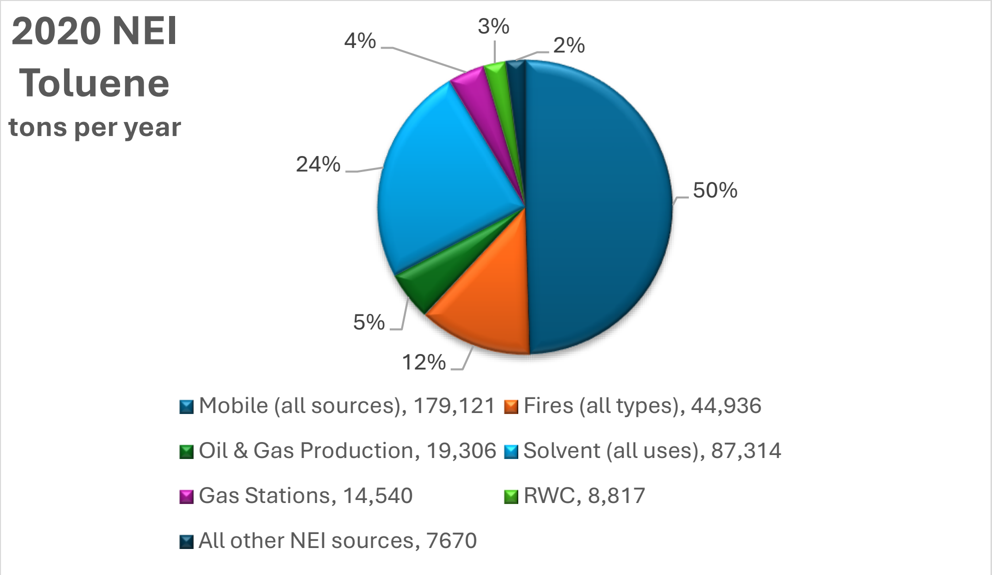

Figure S14: Results of toluene (an EPA HAP) emissions by sector using the 2020 EPA NEI, in tons per year, after applying this study's emissions factors. RWC is the 6th highest source category for toluene in the 2020 NEI.

Figure 15 (a)

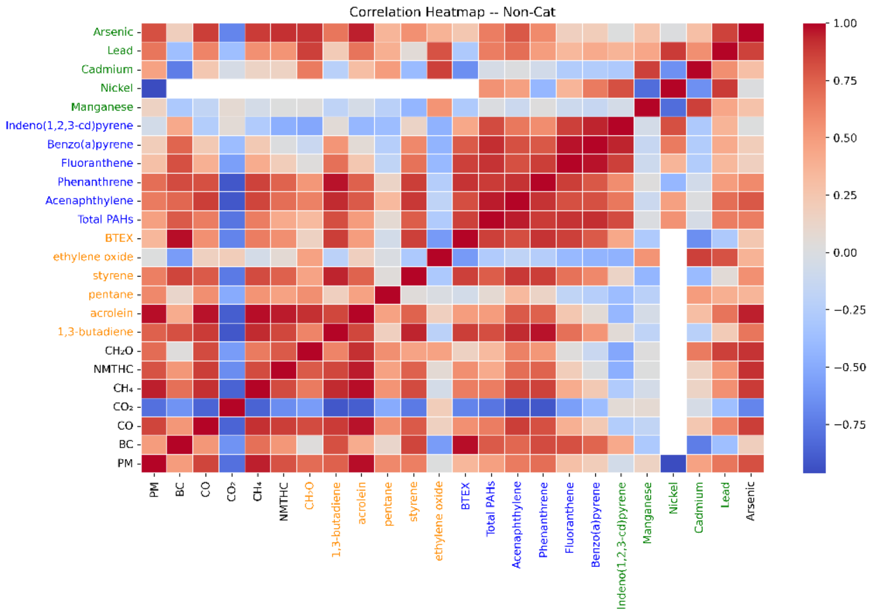
Correlation Heatmap -- Non-Cat
Arsenic
Lead
Cadmium
Nickel
Manganese
Indeno(1,2,3-cd)pyrene
Benzo(a)pyrene
Fluoranthene
Phenanthrene
Acenaphthylene
Total PAHs
BTEX
ethylene oxide
styrene
pentane
acrolein
1,3-butadiene
CH₂O
NMTHC
CH₄
CO₂
CO
BC
PM
1.00
0.75
0.50
0.25
0.00
−0.25
−0.50
−0.75

(b)

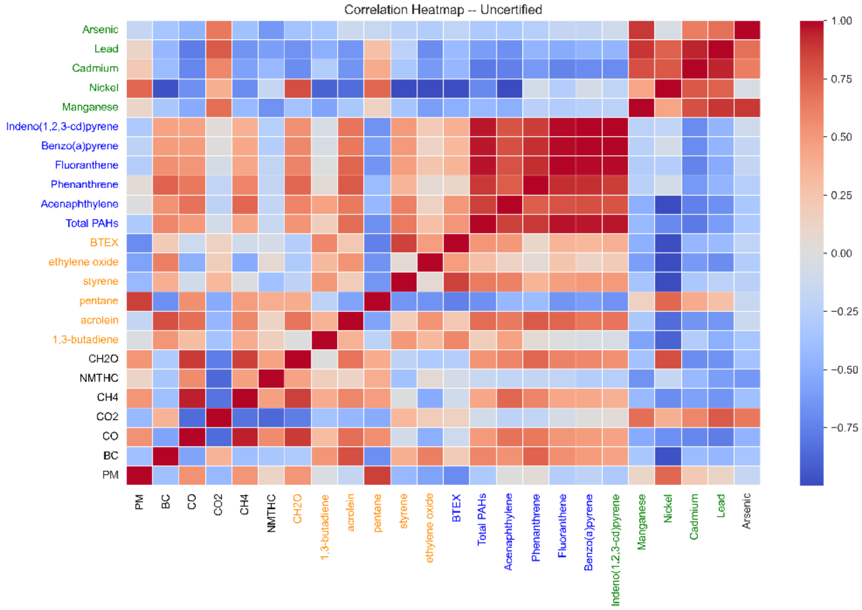
Correlation Heatmap -- Uncertified
Arsenic
Lead
Cadmium
Nickel
Manganese
Indeno(1,2,3-cd)pyrene
Benzo(a)pyrene
Fluoranthene
Phenanthrene
Acenaphthylene
Total PAHs
BTEX
ethylene oxide
styrene
pentane
acrolein
1,3-butadiene
CH2O
NMTHC
CH4
CO2
CO
BC
PM
1.00
0.75
0.50
0.25
0.00
−0.25
−0.50
−0.75

(c)

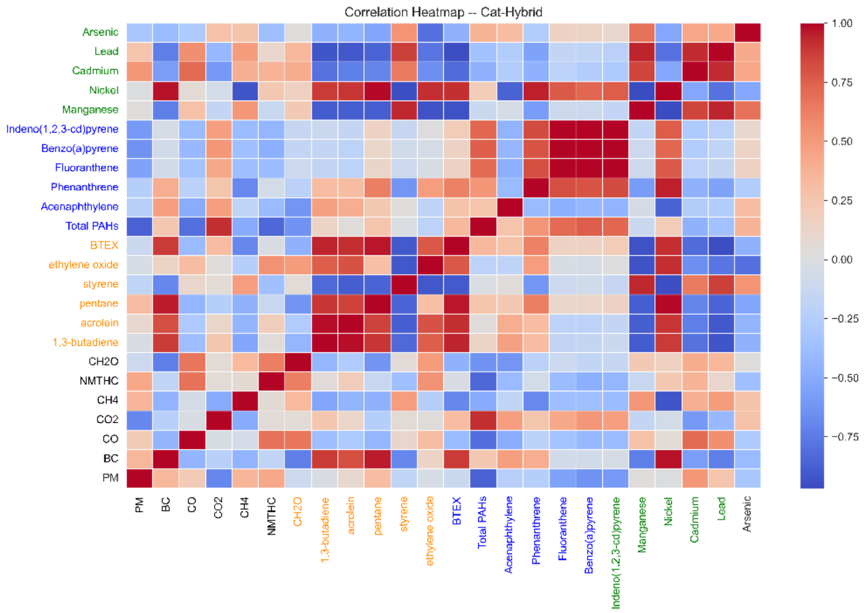

Figure S15 (a), (b), and (c): Correlation heat maps showing relationships between pollutant EFs from non-catalytic (a), uncertified (b) and catalytic/hybrid stoves (c), respectively. Red= positive correlation, and blue = negative correlation. For more detailed EF data per pollutant, see Tables S3 to S14 in Supplemental Information.

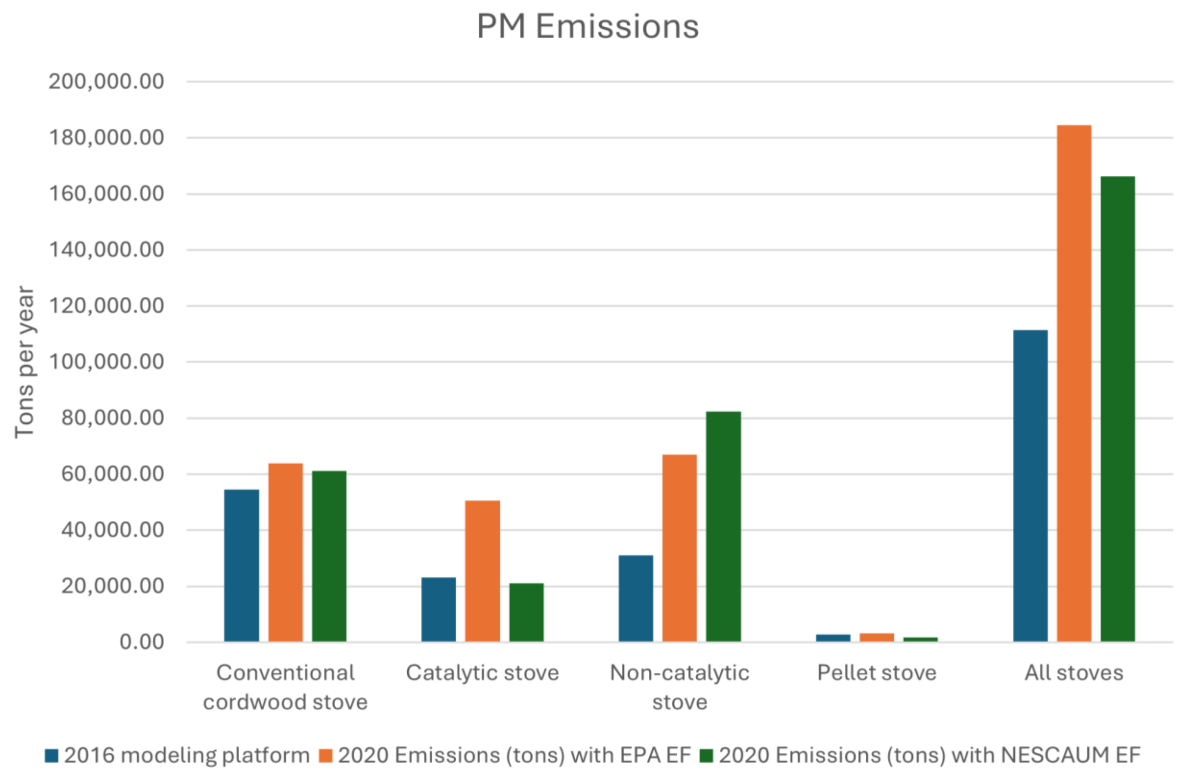

Figure S16: Comparison of national PM emissions from cordwood and pellet stoves using EPA EFs (from the 2016 modeling platform and 2020 NEI) against NESCAUM EFs (2020 NEI), per stove technology category. Applying the NESCAUM EF shows non-catalytic stove PM emissions are underestimated in the 2020 NEI by about 23%, while catalytic/hybrid and pellet stove emissions are overestimated. Conventional woodstove PM emissions are similar between the various inventory years and this study.

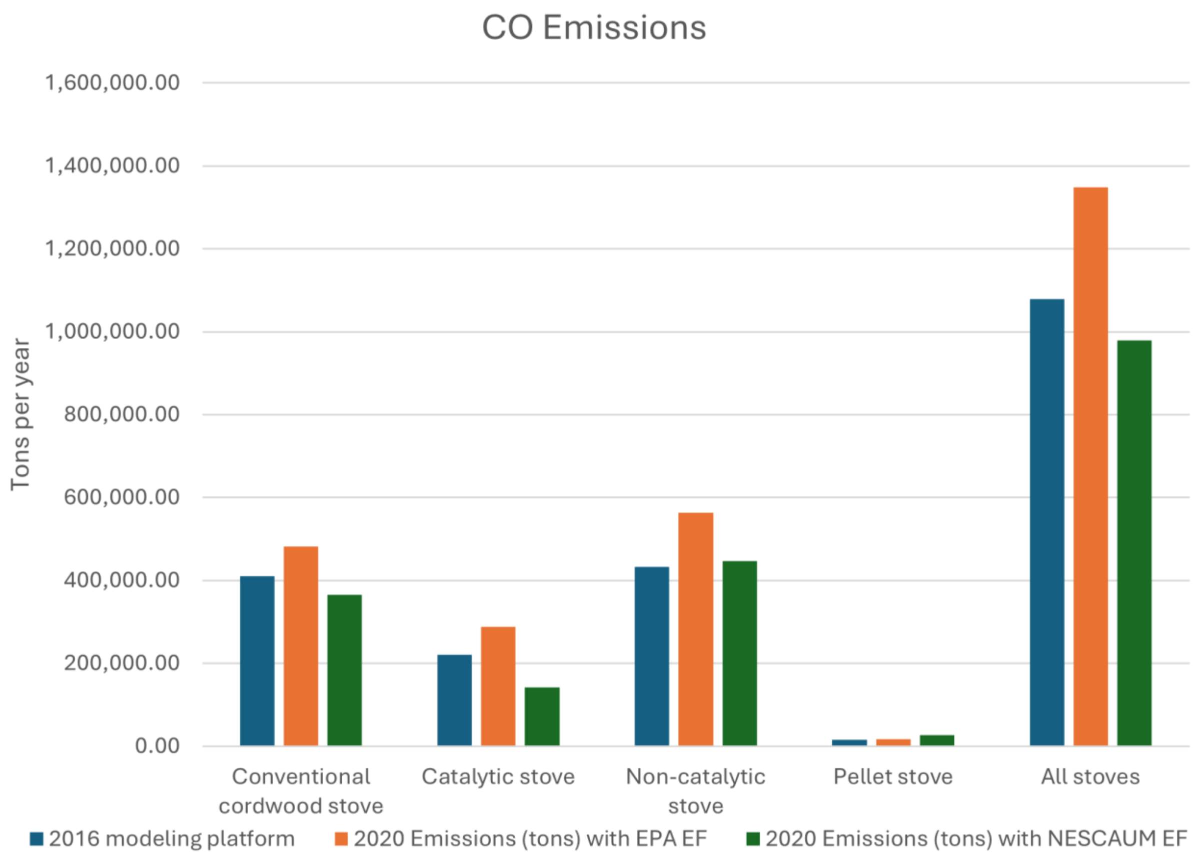

Figure S17: Comparison of national CO emissions using EPA EFs (using the 2016 modeling platform and 2020 NEI) against NESCAUM EFs (2020 NEI), per stove technology category. Applying the NESCAUM EF shows CO emissions are overestimated in the 2020 NEI for all cordwood stove types. CO emissions are underestimated for pellet stoves, with the emissions from applying the NESCAUM CO EF (26,829 tons per year) higher than the 2016 EPA modeling platform CO EF (14,795 tons per year) or 2020 EPA CO EF (16, 407 tons per year).

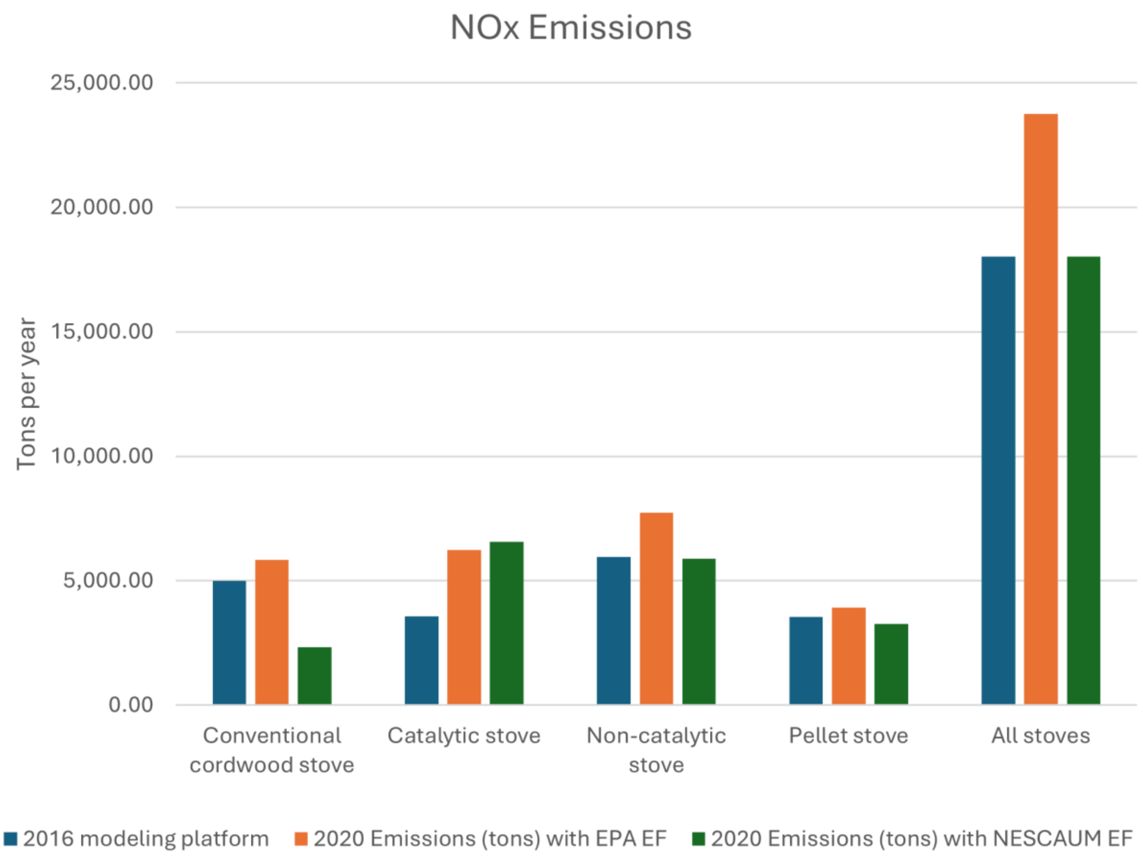

Figure S18: Comparison of national NOx emissions using EPA EFs (using the 2016 modeling platform and 2020 NEI) against NESCAUM EFs (2020 NEI), per stove technology category. Applying the NESCAUM EF shows NOx emissions are overestimated in the 2020 NEI for all stove technologies except catalytic/hybrid. NOx emissions are slightly underestimated for catalytic/hybrid stoves, with the emissions from applying the NESCAUM NOx EF (6555 tons per year) higher than the 2016 EPA modeling platform NOx EF (3558 tons per year) or 2020 EPA NOx EF (6243 tons per year).

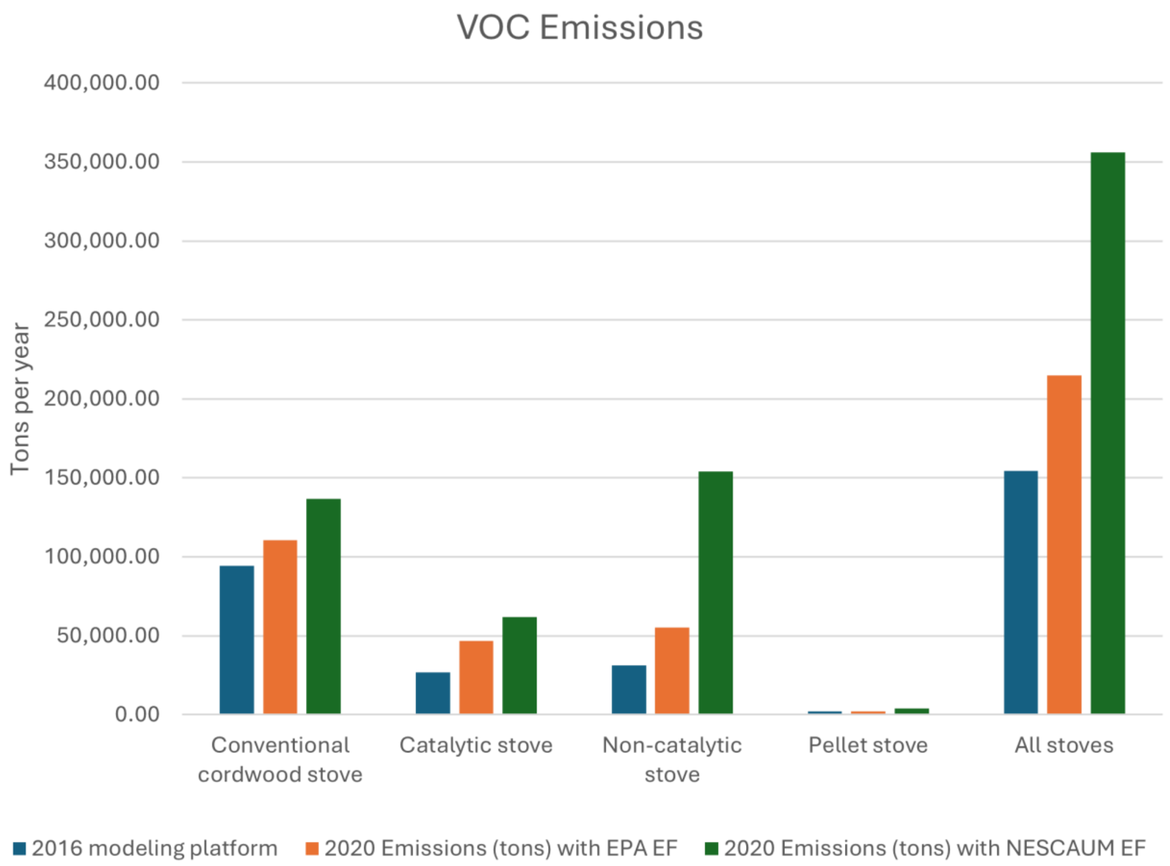

Figure S19: Comparison of national VOC emissions from cordwood and pellet stoves using EPA EFs (from the 2016 modeling platform and 2020 NEI) against NESCAUM EFs (2020 NEI), per stove technology category. Applying the NESCAUM EFs shows VOC emissions from cordwood and pellet stoves are underestimated in the 2020 NEI in all stove technology categories. Specifically focusing on the 2020 NEI comparisons, the largest difference is in the non-catalytic stove category, where VOC emissions are approximately 2.8 times higher using the NESCAUM VOC EF.
